# Conductive hydrogel bioelectronics for chronic neuromodulation: From biomimetic design and bioactive interfaces to integrated neural platforms

**DOI:** 10.1016/j.mtbio.2026.103722

**Published:** 2026-10-07

**Authors:** Chunlin Liu, Yukun Mao, Zhenxing Ren, Yuanhong Shang, Hui Xu, Wanyue Feng, Jixin Zhou, Duo Zhang, Xiang Cui, Xulin Hu

**Affiliations:** a School of Mechanical Engineering, Chengdu University, Chengdu, Sichuan, 610000, People's Republic of China; b Plastic Surgery Hospital, Chinese Academy of Medical Sciences and Peking Union Medical College, Beijing, 100144, People's Republic of China; c College of Biological and Chemical Engineering, Panzhihua University, Panzhihua, 617000, People's Republic of China; d School of Medicine, The Chinese University of Hong Kong, Shenzhen, Guangdong, 518172, People's Republic of China; e Department of Orthopaedics, Chinese PLA General Hospital, Beijing, 100853, People's Republic of China; f National Clinical Research Centre for Orthopaedics, Sports Medicine and Rehabilitation, Chinese PLA General Hospital, Beijing, 617000, People's Republic of China

**Keywords:** Biomedical engineering, Conductive hydrogels, Neuromodulation, Flexible bioelectronics, New approach methodologies, Microphysiological systems

## Abstract

In biomedical engineering, hydrogel bioelectronics brings together the interface between flexible electronic devices and biological systems, opening up a new way for chronic neural regulation. Here we focus on the recent progress in this field, from material design to system integration. The design of high-performance conductive hydrogels is an entry point. The multi-level structure engineering strategy, such as the introduction of carbon nanotubes or conductive polymers, can raise the conductivity and mechanical toughness together. When biomimetic adhesion molecules such as polydopamine or tannic acid are added, the hydrogel has the ability of wet tissue adhesion, and anti swelling and active anti-inflammatory functions are also embedded in the material system. As a result, the resulting neural interfaces are not only more durable in practical applications but also ensure excellent biocompatibility. Turning to applications. How does this kind of material promote the development of chronic neuromodulation technology? Peripheral nerve repair, brain computer interface, spinal cord injury, autonomic nervous system regulation can be seen in several scenes. The long-term and high-precision recording and regulation of neural signals is exactly the most important link in these applications. Hydrogel electronics does have its own uniqueness in this regard. However, there is still a long way to go between laboratory research and clinical transformation. How to ensure long-term stability? Can controllable degradation be achieved in practice? Will the signal fidelity drop with time? How to optimize wireless operation and system integration? Large scale manufacturing is an unavoidable reality test. These directions are probably the worthy areas for follow-up research. Looking forward, if the lines of intelligent response materials, closed-loop sensing and control, and advanced micro processing technology can be screwed together, hydrogel bioelectronics may truly move towards the next generation of personalized, dynamic and adaptive neural diagnosis and treatment platform.

## Highlights

• Conductive hydrogels enable stable, high-fidelity chronic neural interfaces via biomimetic mechanical and bioactive design.
• Hierarchical structural engineering and tannic acid mediation represent two complementary paradigms for electro-mechanical and interfacial optimization.
• Mechano-bioactive hydrogels actively modulate glial responses, opening new avenues for signal fidelity preservation.
• Translational barriers—degradation control, wireless operation, and scalable manufacturing—define the roadmap to clinical adoption.

## Graphical abstract

Hydrogel-Based Neural Interfaces: A Paradigm Shift from Rigid Contact to Active Bio-Integration.

**Figure:**
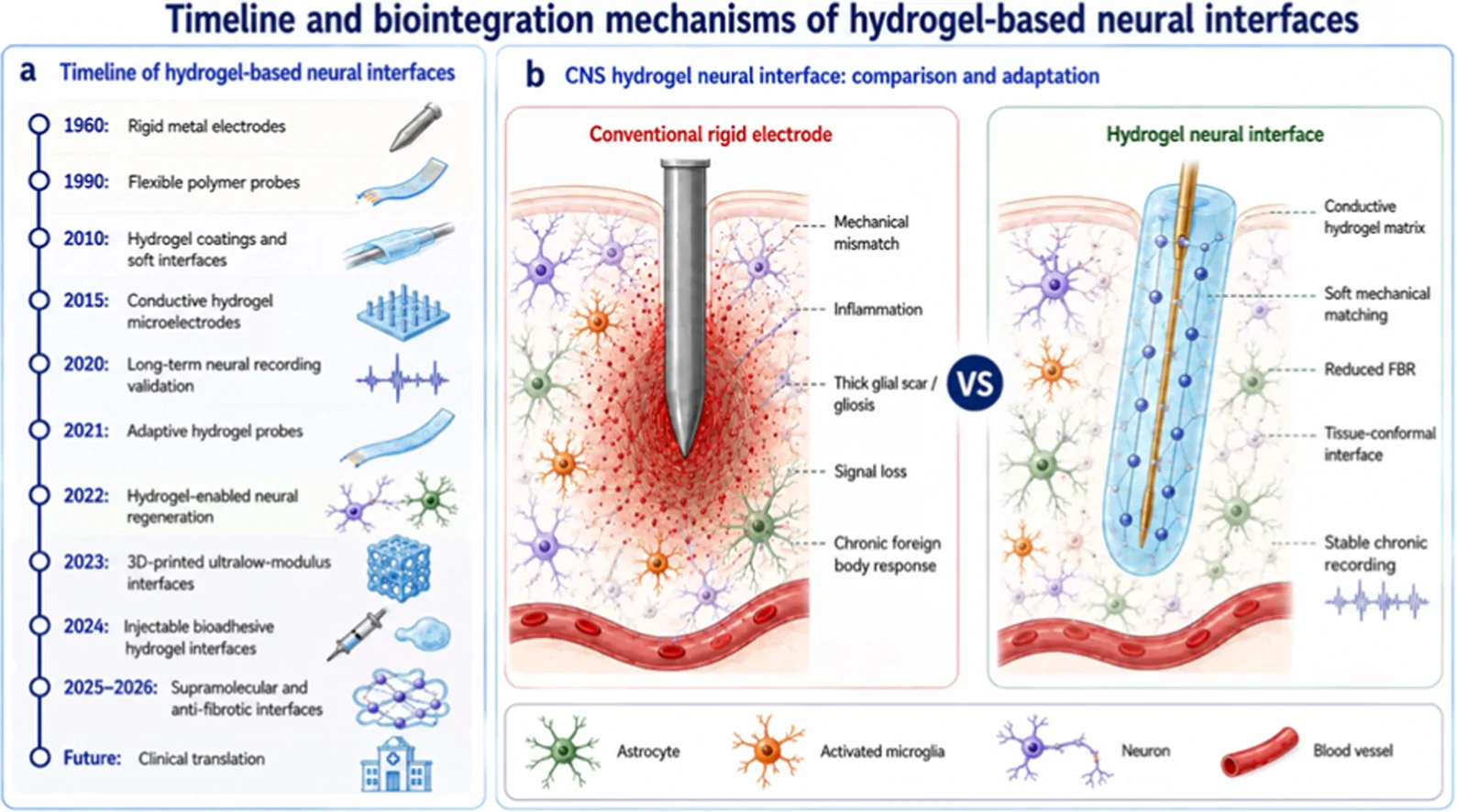

## 1 Introduction

Population aging and the rising prevalence of neurological disorders—including Parkinson's disease, Alzheimer's disease, and refractory chronic pain—are placing mounting pressure on healthcare systems [1,2]. These diseases not only lower the quality of life of patients, but also bring heavy social and economic burden. Systemic drug therapy is often difficult to accurately modulate specific central or peripheral nerve circuits in space and time, and is often accompanied by dose-related side effects. Electrical interventions such as deep brain stimulation can directly act on nerve circuits, but there is a long-term mechanical mismatch between rigid or thin-film electrodes and soft nerve tissue. Fretting, foreign body reaction, colloidal scar, signal attenuation, and even the final device failure is all caused by this. How to solve the mechanical mismatch between tissue and device is still a core problem in the field of chronic neuromodulation (see Table 6).

In this context, the progress of hydrogel materials science provides a feasible path for the construction of mechanically compliant tissue electronic interfaces (Fig. 1). The hydrogel has high water content and adjustable modulus, which can alleviate the mechanical mismatch problem to a certain extent. However, conductive hydrogels cannot be generally classified as a single mixed conduction category [3,4]. The hydrogel, which is dominated by ion conduction, transfers charge by the migration of hydrated ions in water rich pores; In the case of electron conduction, holes, polarons or electrons are transported by the percolation path formed by conjugated polymers, carbon materials, MXene or metal fillers; The ion electron hybrid conductor needs to have both continuous electronic pathways and mobile ion phases involved in charge transfer. The existence of water, electrolyte or electronic filler in the system alone is not enough to prove that the mixed conduction mechanism has been established. Therefore, the classification of this review will be defined according to the verified carrier and conduction path of each system, and the 1 kHz impedance and phase, electrochemical window, charge storage capacity (CSC), charge injection capacity (CIC), noise and signal-to-noise ratio (SNR), cycle stability, hydration aging and other indicators will be used in the device evaluation, rather than the DC conductivity alone [[5], [6], [7], [8], [9], [10]].

**Fig. 1:**
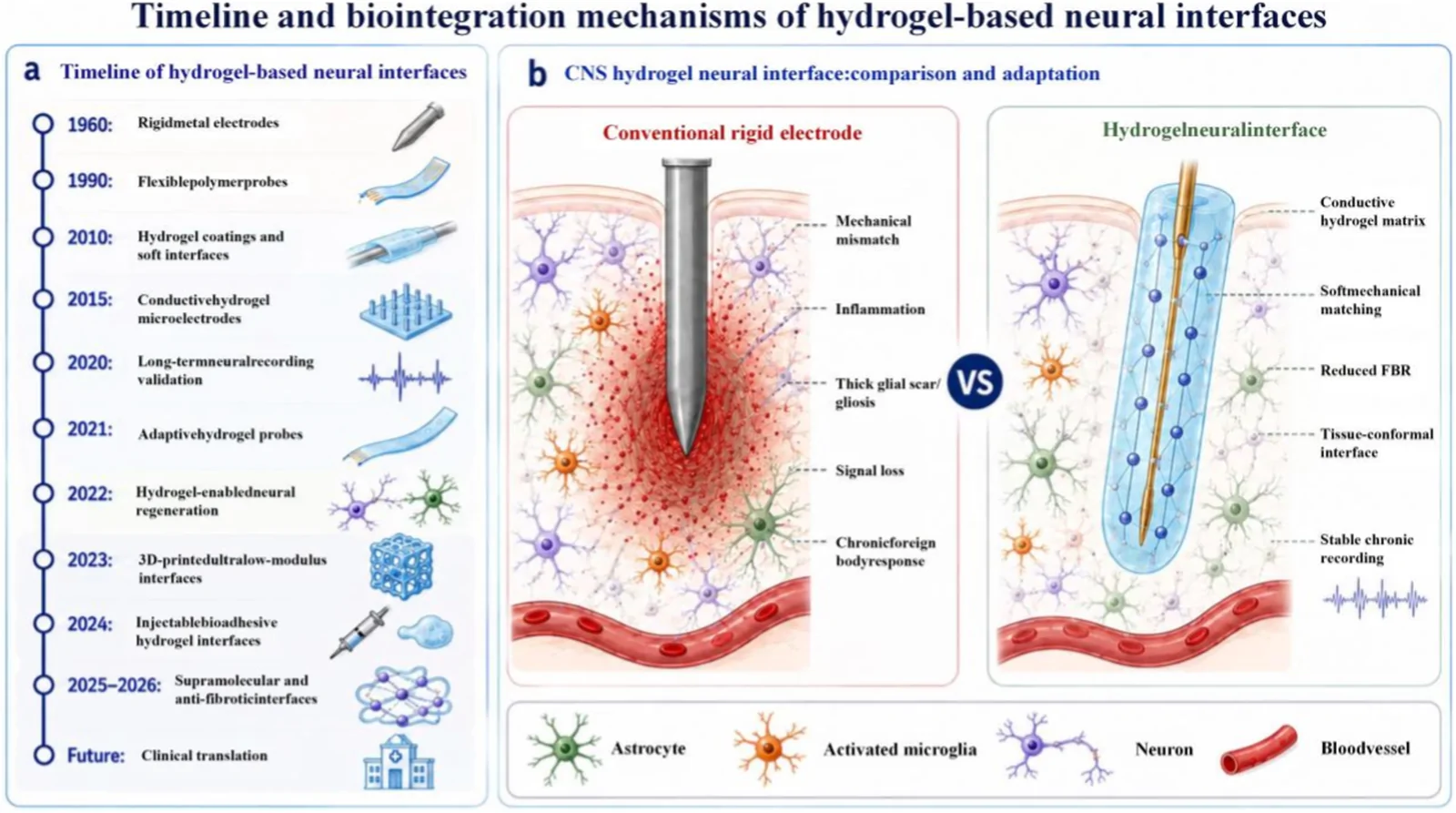
Timeline and biointegration mechanisms of hydrogel-based neural interfaces. Original artwork created by the authors for this review; no third-party figure is reproduced.

Here we define "new methodologies" (NAMs): it refers to non clinical, human related or experimental and computational means that can reduce the use of animals, including 3D culture based on human cells, organoids, organ chip/micro physiological system (MPS), in vitro detection based on mechanism, and computer simulation models. These methods can provide targeted evidence for safety, biological response and device performance [11,12]. MPs is a kind of controlled multicellular in vitro system, which reproduces specific tissue level functions through three-dimensional organizational structure, which can also be combined with perfusion, mechanical loading or electrical stimulation under applicable conditions.

For the conductive hydrogel nerve interface, each model of this framework corresponds to a clear decision node: neural organs can be used for the preliminary screening of formulas and functional materials; Neural chip MPs and targeted toxicity model were used to evaluate local interface toxicity, residual monomers, crosslinking agents, conductive fillers, extractables, and stimulus related damage; Real time and accelerated degradation models help to expose long-term chemical, mechanical, adhesive and electrical failure modes; The computational model can be used to optimize the geometry, current density, thermal effect, strain, swelling and mass transfer before implantation. Therefore, the evidence provided by NAMs can help reduce the number of candidate materials and optimize the species selection, duration, endpoint setting and worst-case conditions of animal experiments. However, at present, it can not replace the human scale surgical operation, systemic immunity and clearance process, as well as the device tissue fretting effect for several years [11,12].

Based on the above progress, conductive hydrogel is gradually becoming the core material of flexible bioelectronic devices, covering brain computer interface, nerve repair catheter, implantable stimulator and other application scenarios (Fig. 2) [13]. This review will first review the materials and interface design principles that the properties of conductive hydrogels depend on, then evaluate the recent research evidence of its chronic regulation on peripheral and central nerve targets, and then analyze the key obstacles for clinical transformation, and try to put forward the priority direction for future development. Later, in section 4.5, we will integrate these considerations into a NAMs driven development and verification path.

**Fig. 2:**
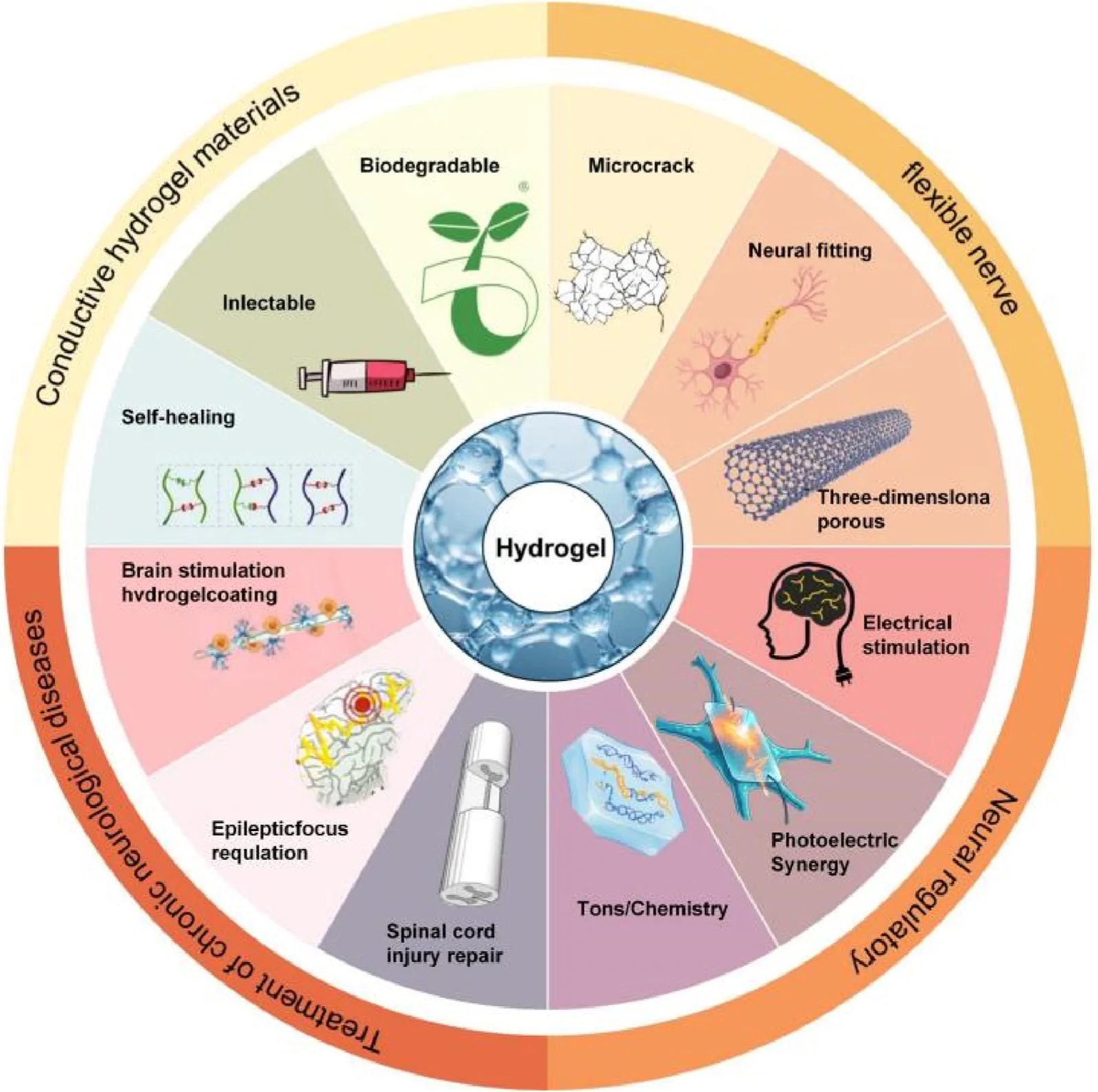
Materials, structural features, and representative application domains of conductive hydrogels in chronic neuromodulation. Original summary schematic created by the authors for this review; no third-party figure is reproduced.

### 1.1 Comparison with recent reviews

Recent reviews have combed the material systems and application scenarios of conductive hydrogels, but their respective concerns are different in question setting methods and evidence units (Table 1). The material centered review focuses more on molecular design, preparation technology and universal bioelectronic functions; The overview of neural interface focuses on the shape and performance of the device; A review of the direction of neural regulation, organizing the content according to the stimulation mode; In the field of tissue engineering, a systematic inventory of regenerative structures is usually carried out. In contrast, we have positioned this review at four levels, which are often separated from each other in previous studies: (I) material and interface design; (II) evidence of chronic recording/stimulation of different targets in the brain, spinal cord and peripheral nerves; (III) manufacturing, sterilization and regulatory readiness; (IV) NAMs driven "model decision" path to reduce and optimize animal experiments [[14], [15], [16], [17], [18]].

**Table 1:** Comparison of representative recent reviews and the distinct organizing contribution of the present review.

Review	Principal scope	Main boundary relative to the present question	Added axis in this review
Zhang et al., 2023 [14]	Conductive hydrogels across bioelectronics; molecular structures, design principles, and operation mechanisms.	Neural targets, chronic in vivo evidence, and clinical/NAM pathways are not the organizing axes.	Cross-target chronic neuromodulation evidence linked to target-specific device and failure metrics.
Xue et al., 2025 [15]	Fabrication, properties, and applications of conductive-hydrogel neural interfaces.	Long-term stability and scalability are discussed, but evidence classes and a model-to-decision NAM pathway are not explicit.	Quantitative preclinical evidence matrix plus manufacturing, regulatory, and NAM decision gates.
Ma et al., 2025 [16]	Hydrogel properties, coatings, electrodes, integrated electronics, and future AI/wireless directions.	Broad technology overview without staged comparison of brain, spinal, and peripheral chronic requirements.	Target-specific surgical and performance requirements tied to chronic evidence maturity.
Zhao et al., 2025 [17]	Five hydrogel-enabled neuromodulation modes and multimodal or closed-loop therapeutic systems.	Translation is discussed conceptually; standardized long-term evidence, regulatory readiness, and NAM validation are not integrated.	A device-centered chain from chronic function and failure to manufacturing, regulation, and NAM validation.
Moghaddasi et al., 2025 [18]	PRISMA-ScR systematic scoping review of 125 conductive nanocomposite-hydrogel studies for neural tissue engineering.	Regeneration and scaffold outcomes dominate; reliability of chronic recording/stimulation devices and regulatory/NAM development are outside the main focus.	A complementary narrative synthesis centered on chronic bioelectronic devices and translational decision-making.

Accordingly, the novelty claimed here is not another catalogue of conductive-hydrogel formulations. It is the use of a device-centered translational framework in which charge-transport and polymer-network architecture are traced through chronic electrical and histological performance, target-specific surgical constraints, manufacturing and regulatory requirements, and finally NAM decision gates. This structure makes it possible to distinguish material promise from evidence that is sufficiently mature to support the next development decision.

### 1.2 Literature search and evidence classification

In order to support a narrative review with transparent structure, we conducted literature retrieval on PubMed, openalex and full-text platforms of various publishers, and supplemented it by reference backtracking and citation tracking. The search was updated on August 26, 2026, mainly covering the period from January 2019 to August 2026; As long as they have established a material principle, a type of device or a long-term neural interface benchmark, the earlier foundational research has also been retained. The core search combinations are ("conductive hydrogel" or "ionic hydrogel" or "hydrogel bioelectronics" or "soft nerve interface") and ("nerve interface" or "neural regulation" or "brain computer interface" or "spinal cord" or "peripheral nerve") and (recording or stimulation or implantation * or chronic or degradation or biocompatibility). Transformation oriented retrieval complements (manufacturing * or sterilization or regulatory or clinical), and NAMs oriented retrieval complements ("new approach methodologies" or organoid or "neural chip" or micro physiology or "computer simulation").

We included the peer-reviewed English original research and review, provided that they involved conductive or ionic hydrogels, hydrogel integrated bioelectronic devices or hydrogel based soft nerve interfaces, and reported at least one of the following related dimensions: composition and charge transport; Mechanical, adhesion, swelling or degradation behavior; Nerve recording or stimulation; Biological response; In vivo implantation; Manufacturing or sterilization; Regulatory transformation; Or NAMs based safety and performance assessment. For the quantitative evidence table, we give priority to the original studies that clearly give the formula, nerve targets and models, implantation duration, and numerical electrophysiological and/or histological endpoints. Wearable devices for non neural applications, non-conductive hydrogels without conductive or interface functions, conference abstracts, patents, repeated reports, and records lacking sufficient methodological details for the claimed conclusions were not included in the core evidence set.

Evidence was classified by study unit, but not statistically combined: E1, evidence of chronic in vivo devices (operationally defined as implantation for at least 4 weeks, accompanied by longitudinal recording or stimulation data, and providing end-point histology when feasible); E2, Acute or short-term in vivo evidence (<4 weeks); E3, Evidence of human related in vitro or NAMs, including nerve cells, organoids, neural chip/mps, targeted toxicity, degradation and computational models; E4, Bench engineering evidence, including electrochemical, mechanical, adhesion, swelling, fatigue and accelerated aging tests; E5, Background evidence from reviews, standards, regulatory guidelines, and equipment databases. These categories indicate the type of evidence and its distance from transformation, while the informal bias risk score. Reviews are used to outline the overall picture of the field, while references to quantitative performance are preferentially traced back to the original research.

Therefore, we position this work as a structured narrative review and evidence map, rather than a PRISMA systematic review. We do not claim to have completed a detailed study count, Prisma flow chart, meta-analysis or merger effect estimation.

## 2 Materials science: design strategies for high-performance conductive hydrogels

### 2.1 Charge-transport framework and conductive-network construction

Classification of charge transport has to be distinguished from the classification of polymer networks. Permanent covalent, dynamic or hybrid polymer networks can carry ions, electrons or mixed transport; On the other hand, the two hydrogels with similar bulk conductivity may have different coupling modes with tissues and electronic hardware. Therefore, the following classification is mainly based on the dominant mobile carriers, continuous transmission paths, and existing electrochemical evidence. If the original study did not clarify the respective contributions of ionized ions and electrons, we would only describe the dominant type, rather than give it an absolute label without basis.

#### 2.1.1 Predominantly ionic conduction

The conductivity of hydrogel, which is dominated by ion conduction, depends on the migration and diffusion of hydrated cations and anions driven by electric field in the aqueous phase. The charge on the fixed polymer chain, ion pair Association, hydrogen bond and reversible metal coordination will be involved, which will affect the ion concentration and mobility; In addition, dehydration or electrode polarization may also significantly increase the resistance. Taking CMCNa/PAA/Fe^3+^ hydrogel as an example (Fig. 3b), Fe^3+^ coordination and hydrogen bond mainly play the role of stabilizing and dissipating energy in the polymer network; The bulk current is mainly carried by mobile counter ions and other dissolved ions in the aqueous phase [19]. This paper reported the strain sensing based on resistance change, but did not provide the impedance and phase normalized by area at 1 kHz, clear electrochemical window, csc/cic data, recording noise or signal-to-noise ratio, nor the experimental results of long-term immersion at 37 °C. Therefore, it is more suitable to be regarded as evidence at the level of ion conducting materials, and is not enough to constitute a complete characterization of nerve electrodes.

**Fig. 3:**
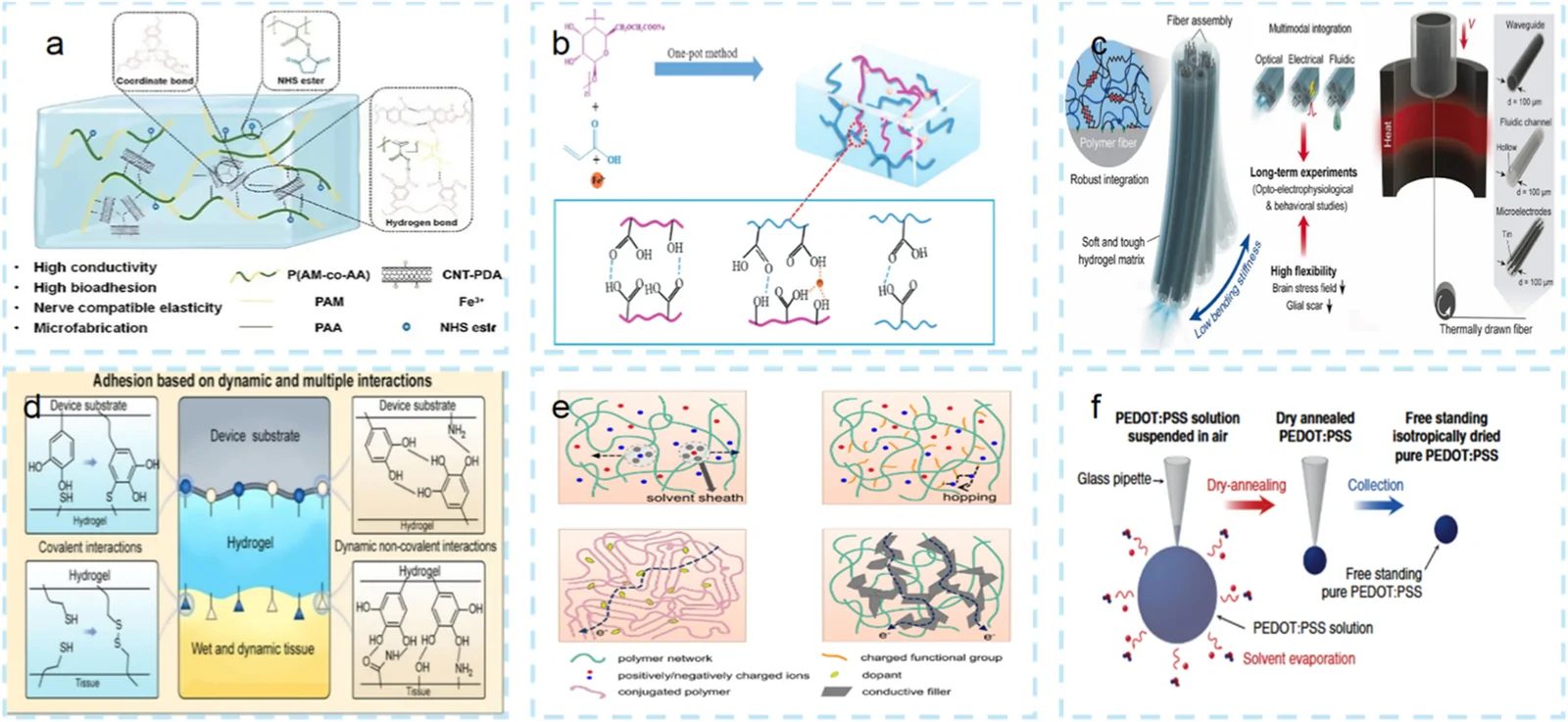
(a) PAACP conductive-hydrogel architecture. Copyright 2025, American Chemical Society. (b) Preparation and ionic-network mechanism of the CMCNa/PAA/Fe^3+^ hydrogel. Copyright 2019, MDPI AG. (c) Adaptive multifunctional hydrogel hybrid probe for long-term neural sensing and modulation. Copyright 2021, Nature Publishing Group (Springer Nature). (d) ICAA adhesion to tissue and device substrates through click chemistry and multiple dynamic non-covalent interactions. Copyright 2024,Springer Nature. (e) Schematic ion transport through a neutral, water-rich three-dimensional polymer network. Copyright 2024, Springer Science and Business Media LLC (Springer Nature). (f) Fabrication of free-standing pure PEDOT:PSS hydrogels through dry annealing and subsequent rehydration of aqueous PEDOT:PSS. Copyright 2019, Springer Nature.

#### 2.1.2 Predominantly electronic conduction

The premise of electron conduction is that there is a percolation path in the system that allows electrons, holes or polarons to pass through. Let's start with t-ech. The PAA template plays a role of confinement and ordering, which makes the p-doped PEDOT:PSS form a nanofiber network; Holes and polarons shuttle within and between chains along the continuous region of PEDOT enrichment, and PSS is responsible for stabilizing the doped state [20]. Hydration provides an electrolyte environment for interfacial electrochemistry, but does not change the electron dominated attribution in the bulk phase. The conductivity of t-ech reaches 247 s cm^−1^, the 1 kHz impedance of PBS is 27.8 Ω, and the CSC reaches 80 MC cm^−2^. Its cyclic voltammetry scanning range is set at − 0.5 to+0.5 v. the impedance and CSC in PBS can be stable for 7 days, and the conductivity in water or PBS can remain unchanged for 35 days. However, it should be pointed out that the signal-to-noise ratio of 60.08 dB in the report is from epicardial ECG, not neural records; The exact phase angle and CIC of 1 kHz are also not given in numerical form [20].

Pure PEDOT:PSS hydrogels provide a representative example of predominantly electronic conduction. As illustrated in Fig. 3f, aqueous PEDOT:PSS can be converted into a free-standing hydrogel through dry annealing followed by rehydration. The interconnected PEDOT-rich nanofibrillar domains provide the principal electronic transport pathway, whereas the hydrated PSS-rich phase facilitates ionic exchange at the electrode–electrolyte interface.

PPy/aramid nanofibers/pva hydrogels also belong to the electronic dominant category. Polarons migrate in the three-dimensional percolation network composed of PPy nanofibers, while aramid nanofibers and PVA are responsible for supporting mechanical loads [21]. The conductivity of about 80 s cm^−1^ and the tensile strength of 9.4 MPa indicate that electron percolation has indeed been established, but the water content alone is not enough to prove that it is a mixed conductor in the quantitative sense. In this study, the patterned electrode was characterized by electrochemical impedance and charge injection, but the values of 1 kHz phase angle, recording noise/signal-to-noise ratio and several weeks immersion stability were not directly given in the text, and should not be inferred only by conductivity.

After PPy, let's look at CNT-pda. In paacp hydrogel, CNT-pda formed a one-dimensional electron percolation skeleton in pam-paa matrix (Fig. 3a); The polydopamine coating improves the dispersion and interface bonding of CNTs, and catechol/NHS chemistry contributes to adhesion function rather than additional electronic pathways [22]. The conductivity of 9.52 s m^−1^ is sufficient to indicate that it is an electron dominated composite material. Physiological ions may indeed migrate in the aqueous polymer phase, but the number of ion migration has not been measured, so it is not suitable to directly label paacp with mixed conduction.

On the TN-H side, acid treated PEDOT:PSS plays the role of electronic pathway, while PEGDA and entangled peg provide mechanical properties and adhesion [23]. Its impedance at 1 kHz is 91.2 Ω cm^2^, and the phase angle response is shown in the figure, but the exact value of 1 kHz is not explained in words. In the CV scanning range of − 0.5 to+0.5 V, the CSC reached 46.50 MC cm^−2^ and the CIC was 283 μC cm^−2^. When the strain was increased to 100%, the impedance amplitude was less than 0.4 times, the CSC remained 85%, and the CIC was only slightly fluctuating; However, the data of noise/signal-to-noise ratio and electrochemical immersion stability for several weeks have not been disclosed [23].

#### 2.1.3 Mixed ionic-electronic conduction

To establish a mixed ion electron conductor, two things must be possessed at the same time: one is a continuous electronic network, and the other is an ion phase that can move freely. Moreover, the coupling between ions and electrons can not only be a surface effort, either through the whole material, or at least on the inner interface with high specific surface area. When ICAA is examined, it is indeed called mixed conduction at the interface of hydrated bioelectrode. Electrons and holes percolate through the MXene/PEDOT:PSS/ta network, while ions migrate through the PBS filled peg network, playing a role of charge compensation in impedance measurement and biphasic pulse test [24]. The bulk conductivity of ICAA was 92.43 ± 7.65 s m ^−1^, and the 1 kHz impedance was maintained at about 120 Ω after soaking in PBS at 37 °C for 4 weeks. CIC was 139 μC cm^−2^, which changed little after 100000 charge discharge cycles, and remained stable during 4 weeks of immersion; The conductivity is maintained at about 95 s m ^−1^. However, the accurate phase angle of 1 kHz, the CSC value, the fully verified electrochemical stability window, and the recording noise/signal-to-noise ratio have not been seen in the literature. In addition, the migration numbers of ions and electrons have not been measured, so the relative contribution of the two in the bulk phase cannot be quantified, although the ion electron conversion process at the device level has indeed been verified.

#### 2.1.4 Material-to-device metrics

In the final analysis, bulk conductivity is only a descriptive index at the material level, and it is still far from being used to predict recording or stimulation performance. At least, the following aspects should be covered in the data report of neural interface research: (I) impedance amplitude and phase angle at 1 kHz, and the geometric area, area normalization method, electrode configuration, disturbance amplitude, bias condition, medium and temperature should be noted; (II) electrochemical stability window - it shall be defined based on oxygen evolution/hydrogen evolution or irreversible reaction, and shall be clearly distinguished from the scanning range of cyclic voltammetry; (III) CSC from CV and CIC from charge balance pulse, with waveform, pulse width, voltage/current limit and polarization criteria attached; (IV) input reference noise or electrode noise (root mean square voltage and/or power spectral density), and signal-to-noise ratio - the signal-to-noise ratio needs to give a clear signal definition and bandwidth; (v) The number of electrical and mechanical cycles, amplitude, frequency, and impedance/csc/cic retained after the cycle; (VI) long term immersion data in PBS or serum at 37 °C. impedance, phase angle, conductivity, swelling, mass loss, adhesion and electrochemical indicators were measured at matching time points.

Looking back at the current evidence, we can only say that there are different emphases, but they are not complete. T-ech provides 1 kHz impedance, CSC, CV scanning range, ECG SNR and short-term hydration stability, but it lacks the value of 1 kHz phase angle and cic [20]. TN-H gives 1 kHz impedance, phase angle (in graphical form), CV range, csc/cic and retention rate under strain, but the recorded noise/signal-to-noise ratio and weeks of immersion data have not been reported [23]. ICAA took out 1 kHz impedance CIC, 100000 cycles stability and 4-week PBS aging data, but lack of phase angle value CSC, The stability window and noise/signal-to-noise ratio are clearly verified [24]. As for paacp, PPy/ANF/PVA and ionic CMCNa/PAA/Fe^3+^ systems, those unreported items are actually recorded as NR, which cannot be inferred from bulk conductivity or other device demonstrations [19,21,22]. Adhering to this reporting specification can at least avoid mistaking the conductivity at the material level for a fully validated chronic nerve interface.

### 2.2 Self-adhesion and biocompatibility

Hydrogel based bioelectronic devices in order to function reliably in chronic neuromodulation, we need to cross a barrier - how to establish a stable, mild and lasting interface integration with dynamic neural tissues. The ideal interface should be sutured free and firmly attached, so that the surgical trauma can be minimized, and stable interfacial contact can be maintained in the physiological fluid environment [[25], [26], [27], [28], [29], [30], [31], [32], [33], [34], [35], [36], [37], [38]]. In this way, the water gel itself is equipped with the ability of wet tissue adhesion, and at the same time, the biocompatibility has become a cutting-edge issue in material design [[29], [30], [31]].

Bionic strategy and precise molecular design have brought some noteworthy progress on this road. Using exfoliated graphite as conductive filler, slow gelling (SG) hydrogel was prepared by microwave irradiation polymerization, and a viscoelastic network structure with distinct mechanical characteristics was constructed. This synthetic route allows the mechanical properties of hydrogel - modulus and relaxation behavior - to be adjusted in a relatively fine range, so as to match the mechanical characteristics of biological soft tissue. In short, depending on the tissue like viscoelasticity and moderate surface viscosity of the material itself, it can be tightly attached to the nerve adventitia by physical action without any chemical adhesive [32]. This kind of bonding is not violent, but it is stable enough to maintain the interface contact for several weeks, and the signal attenuation and tissue damage caused by movement are also suppressed. Long term peripheral nerve recording and regulation, finally has a reliable interface foundation.

To improve the interface stability of hydrogel in complex physiological environment and add ideal biological activity to it, researchers have focused on multifunctional natural molecules. Take ICAA-C hydrogel, which is injectable, conductive, adhesive and anti swelling. As soon as the polyphenolic tannic acid (TA) comes in, the material properties begin to change synergistically. TA carries catechol groups and pyran rings, and can interact with polymer chains and proteins/polysaccharides on the surface of nerve tissues in a variety of non covalent ways-hydrogen bonds, hydrophobic interactions, cationic - π interactions, and so on [31,34]. This molecular design not only gives the hydrogel good wet biological adhesion, but also limits the hydration swelling due to the increase of network density caused by crosslinking, which makes the long-term stability of mechanical matching more confident. In addition, Ta has the characteristics of anti-oxidation and anti-inflammatory, so the biocompatibility of the material is also affected. High conductivity, anti swelling, strong adhesion and biocompatibility, which are integrated within a single material system, the built bioelectronic interface is relatively stable in most cases, at least providing a practical material basis for the chronic precise regulation of key targets such as the vagus nerve [24,33].

The development of self-adhesive conductive hydrogel has gone beyond the simple functional positioning of traditional tissue glue. It integrates bionic adhesion chemistry and swelling control to form a fused material tissue interface. The goal is clear: to stabilize the coupling of mechanical compliance and function under wet and dynamic conditions. The adaptive hydrogel probe shown in (Fig. 3c) just illustrates the role of the flexible interface in maintaining conformal nerve contact. However, the conformal property alone is not enough to prove that the wet adhesion or anti swelling properties are in place-these properties need to be verified one by one under relevant physiological conditions by special adhesion test and swelling measurement [28,[35], [36], [37]].

### 2.3 Comparison of material design paradigms: from bulk properties to interfacial integration

The paacp and ICAA hydrogels discussed in sections 2.1, 2.2 represent two complementary strategies, the bulk network and the injectable interface, respectively. Therefore, Table 2 not only lists the key performance values, but also gives the tested formula, medium, temperature, test method, sample size and statistical presentation. Any item that cannot be verified from the cited original study is directly marked as "unreported" (NR).

**Table 2:** Comparison of design strategies and key performance metrics for two representative conductive hydrogels.

Metric	PAACP [22]: reported result	PAACP [22]: test conditions and reporting	ICAA [24]: reported result	ICAA [24]: test conditions and reporting
Composition/tested formulation	PAM-PAA matrix containing 0.33 wt% CNT-PDA; catechol- and NHS-ester-mediated adhesion.	Formulation identity; no test medium or temperature applies. Reporting: NA	PEG-8SH (15 wt%) and PEG-2Mal (10 wt%) network; MXene (4 wt%), PEDOT:PSS (PH1000 dispersion), and TA (0.3 wt%); equal-volume mixing at pH 7.4.	Formulation identity; no test medium or temperature applies. Reporting: NA
Electrical conductivity (S m^−1^)	9.52	Bulk conductivity measurement; hydration medium, temperature, and full instrument/protocol were NR with the tabulated value. Reporting: Plotted data were presented as mean ± SD; exact SD and n for 9.52 S m^−1^ were NR in the main-text report.	92.43 ± 7.65	Fully swollen in 1× PBS; four-point probe (KDY-1). Baseline measurement temperature NR; long-term immersion tests used 37 °C. Reporting: n = 5 independent experiments; mean ± SD; one-way ANOVA followed by Tukey post hoc test for group comparisons.
Tensile modulus (kPa)	Approximately 100	Uniaxial tensile test using a universal testing machine (CMT6103); medium, temperature, specimen dimensions, and loading rate NR with the summarized value. Reporting: Approximate value; dispersion and n NR.	32.3 ± 2.9	Equilibrated in 1× PBS; CTM8000 universal testing machine; 25 × 5 × 1.5 mm specimens; 20 mm min^−1^; test temperature NR. Reporting: n = 3 independent experiments; mean ± SD; one-way ANOVA followed by Tukey post hoc test.
Wet-tissue adhesion strength (kPa)	107.14	Adhesion to pigskin; exact hydration medium, temperature, specimen geometry, and loading protocol NR with the summarized value. Reporting: Mean ± SD was used for plotted data; exact SD and n for 107.14 kPa were NR in the main-text report.	20.9 (nerve tissue)	Lap-shear test on nerve tissue; 10 min curing at 37 °C in a humid environment; 20 mm min^−1^; overlapping area 18 × 1.7 mm. Reporting: n = 3 independent experiments; mean ± SD in the source figure; the text reports 20.9 kPa without the exact SD; ANOVA/Tukey.
Swelling/anti-swelling	Not reported	No quantitative swelling test was reported. The former >300% entry describes tensile deformation on Ecoflex, not swelling, and was removed. Reporting: NR	Equilibrium swelling ratio: 9.22 ± 0.76%; equilibrium reached within 24 h.	Gravimetric mass-change method in 1× PBS at 37 °C; monitored for 96 h; SR = (Wt - W0)/W0. Reporting: n = 3 independent experiments; mean ± SD.
Antioxidant activity (DPPH/ABTS or equivalent)	Not reported	No directly comparable antioxidant assay was reported. Reporting: NR	Not reported	TA was a formulation component, but no DPPH, ABTS, or equivalent quantitative antioxidant assay was reported. Reporting: NR
In vivo inflammatory/histological response	No directly comparable Iba-1 endpoint identified for this table	Sciatic-nerve repair was evaluated in vivo, but it should not be merged with an antioxidant assay or treated as directly comparable to the ICAA cuff histology. Reporting: NR for a directly comparable endpoint.	After 4 weeks, Iba-1 did not differ significantly from sham; tissue damage, fibrosis, and inflammation were lower than with the sutured cuff control.	Rat cervical vagus nerve; ICAA-C versus 300 μm sutured cuff and sham; H&E and immunofluorescence for Iba-1, S-100, and NFM; ImageJ quantification. Reporting: n = 5 animals per group; mean ± SD; one-way ANOVA followed by Tukey post hoc test.
Device-level electrical stability	Not reported under a standardized long-term PBS protocol	A directly comparable multiweek immersion protocol with conductivity, impedance, and charge-injection readouts was NR. Reporting: NR	Approximately 95 S m^−1^ conductivity and approximately 120 Ω impedance at 1 kHz after 4 weeks; CIC 139 μC cm^−2^ and negligible change over 100,000 cycles.	1× PBS at 37 °C for 4 weeks; four-point conductivity; three-electrode EIS (0.1–100,000 Hz, 5 mV) and biphasic charge injection (1.5 ms, ±0.5 V). Reporting: n = 5 independent experiments for long-term electrical measurements; mean ± SD; ANOVA/Tukey where specified.

For paacp, the 9.52 s m-^1^ conductivity, tensile modulus of about 100 kPa and tissue adhesion strength of 107.14 kPa given in literature [22] do show the balance between electrical mechanical properties and adhesion function. However, this document does not provide quantitative swelling test data for this formula. The ">300%" in the previous table is actually a tensile demonstration from ecoflex support, which is not the same as the swelling ratio - so we deleted this data in Table 2 and changed to "unreported".

We reserved the conductivity, tensile modulus, adhesion and swelling value of ICAA, but strictly limited their test conditions [24]. The equilibrium swelling ratio of 9.22 ± 0.76% was measured by weighing method at 37 °C and 1 × PBS; The results of tissue response were obtained from the experiment of vagal nerve implantation in rats for 4 weeks. Because antioxidant test and histological inflammation endpoint are not the same thing, Table 2 lists "antioxidant activity" and "in vivo inflammation/histological response" separately. As for the determination results of DPPH or ABTS, no relevant data were found in the cited literature.

#### 2.3.1 Conduction and network design paradigm: a higher level comparative analysis

Table 2 gives a detailed comparison of two representative systems. Looking at these two sets of data alone, we can see some differences, but it is not enough to rely on them to establish common design criteria. From a broader perspective, the performance of conductive hydrogels depends on two relatively independent dimensions: one is the charge transport architecture, including nanocomposites, conductive polymers, ion conduction, electron conduction and ion electron mixed conduction; The other is polymer network architecture, which is divided into permanent covalent, dynamic and hybrid. Therefore, when comparing different systems, we should take into account the electrical properties, mechanical behavior, long-term stability, cell compatibility, processability and manufacturing burden, not just the initial conductivity.

The first four rows of Table 3 are classified by charge transfer architecture, and the last two rows are classified by polymer network architecture. These two sets of classification methods complement each other, not mutually exclusive. For example, carbon nanotube composite systems or PEDOT:PSS hydrogels can also be built on permanent covalent, dynamic or hybrid polymer networks. Since most original studies did not report device-level manufacturing costs using a consistent metric, we do not perform quantitative ranking here.

**Table 3:** Comparative analysis of major conductive-hydrogel design paradigms for chronic neural interfaces.

Design paradigm	Conductivity and mechanical profile	Chronic stability and cytocompatibility	Processability, cost, and representative evidence
Carbon-nanomaterial composite hydrogels (CNT, graphene/rGO)	Electronic percolation can provide high conductivity and, with good filler-matrix coupling, mechanical reinforcement.	Performance depends on dispersion and preservation of percolation during hydration and cyclic deformation. Medium-term neural studies can show favorable tissue responses, but long-term redistribution, release, and biological fate remain incompletely resolved.	Scale-up is sensitive to nanomaterial dispersion, aggregation, and surface functionalization. Device-level cost is rarely quantified [22,39].
Conducting-polymer hydrogels (PEDOT:PSS; PPy/PANI-based systems)	Low interfacial impedance and high electronic conductivity are achievable; optimized PEDOT:PSS hydrogels can also provide stretchability and wet stability.	Chronic behavior depends on hydration, dopant/network organization, oxidation, and interfacial adhesion. A four-month coating study found better chronic recording stability for PEDOT/CNT than PEDOT:PSS, showing that initial impedance alone is not predictive.	Solution processing and advanced fabrication, including 3D printing, are feasible for optimized systems. Standardized material or device cost is not reported [[40], [41], [42]].
Predominantly ionic conductive hydrogels	Hydrated ions carry charge through compliant water-rich networks, providing tissue-like mechanics and ionic coupling.	Ion exchange/redistribution, dehydration, and excessive swelling can change impedance over time; cytocompatibility remains formulation- and salt/additive-dependent.	Casting, molding, and injection are generally accessible, but water management, packaging, and storage are critical. Quantitative cost comparisons are lacking [43,44].
Mixed ionic-electronic architectures	Simultaneous ionic and electronic transport can reduce the charge-conversion mismatch between tissue and electronic hardware while retaining soft mechanics.	Ionic and electronic phases may age on different time scales; hydration and morphology changes can alter coupled transport and interface impedance. Safety depends on both phases and possible leachables.	Printing and multilayer integration are possible, but multicomponent control increases manufacturing complexity. No standardized cost comparison is available [40,41].
Permanent covalent networks	A permanent polymer skeleton provides dimensional stability and can suppress excessive swelling when crosslink density is appropriately designed.	Irreversible bond rupture is not intrinsically self-healing; without dissipative or anti-fatigue mechanisms, cyclic damage can accumulate. Safety depends on crosslinker chemistry and residual monomer removal.	Chemistry can be reproducible, but crosslink density must be tightly controlled because it affects modulus, swelling, and diffusion simultaneously. Cost is rarely quantified [32].
Dynamic or covalent-dynamic hybrid networks	Reversible bonds dissipate energy, permit stress relaxation, and can support self-recovery; hybrid networks can combine a stable skeleton with adaptive interactions.	Time-dependent bond exchange can introduce creep or hysteresis if insufficiently constrained. Formulation-specific ligand/ion safety and long-term leaching must be evaluated; hybrid design can improve dimensional stability.	Mild gelation and injectability can aid processing, but reaction kinetics and environmental sensitivity complicate process control and sterilization validation. Cost is process-dependent and rarely quantified [24,32].

Carbon nano material composite system and conductive polymer hydrogel just illustrate a problem: the high initial conductivity does not mean that the chronic performance must be reliable. CNT or graphene-based hydrogels can form an electron percolation network and enhance the polymer matrix. However, whether their long-term electrical properties are good or not depends on whether the filler is dispersed evenly enough, whether the interface between filler and matrix is firmly bonded, and whether the seepage path can be maintained during repeated deformation and hydration. An eight-week study of multilayer graphene hydrogel film on rat sciatic nerve showed that the inflammatory reaction was not obvious, which provided a certain degree of medium-term safety evidence. However, the redistribution, release and clearance of carbon nanomaterials after long-term implantation are still pending [39]. The optimized PEDOT:PSS hydrogel can maintain a stable conductivity for several months in the wet state, while the bicontinuous conductive polymer hydrogel has a conductivity of more than 11 S cm-^1^, a tensile ratio of more than 400%, and also has three-dimensional printability [40,41]. A four-month intracortical comparative study further found that although the initial impedance of PEDOT/CNT and PEDOT:PSS coatings was low, their chronic recording stability was not better [42]. Although the work used coating rather than bulk hydrogel, it directly explained a truth: no matter how good the initial conductivity or impedance is, it cannot be used alone to predict the long-term performance of the nerve interface.

There are similar trade-offs between permanent covalent networks and dynamic networks. Covalent framework can maintain geometric configuration and dimensional stability, but once the bond breaks, it is difficult to repair by itself, and it is usually necessary to introduce additional energy dissipation mechanism to obtain high toughness and fatigue resistance [32]. Reversible hydrogen bonding, coordination and other dynamic crosslinking can dissipate energy and adapt to tissue movement, but if the bond exchange is not sufficiently constrained, stress relaxation, creep or hysteresis may appear. The practical significance of these characteristics depends on the target anatomical site and the loading history it has experienced. ICAA provides an example of hybrid thinking: the stable peg network is matched with the reversible interaction in MXene/PEDOT:PSS/TA conductive network. This system controls the equilibrium swelling ratio at 9.22 ± 0.76%, and the conductivity in PBS is maintained at about 95 s m^−1^ after four weeks, indicating that the dynamic adaptability and dimensional/electrical stability can be decoupled through network engineering to a certain extent [24].

From these comparisons, we can sort out several unsolved problems. First, hydrogels containing CNT or graphene have good short-term cellular compatibility, but the long-term biological distribution and final destination of the released nanomaterials are still unclear. Second, conductivity and swelling are often coupled together-increasing ions or hydrophilic components is conducive to charge transfer, but may also push up water absorption; Network design can decouple the two to a certain extent [24]. Third, even if the initial electrical performance is good, the chronic signal quality is sometimes dominated by tissue response, interface adhesion, hydration state, interconnection and packaging [42]. Fourth, reversible bonding improves energy dissipation and self recovery, but may introduce time-varying deformation. Fifth, although ultra-low modulus can reduce mechanical mismatch, it makes implantation, positioning and geometric stability more difficult. Based on these, long-term research should focus on impedance, signal-to-noise ratio, channel survival, swelling, fatigue, material loss and quantitative histological data, rather than just focusing on the peak indicators of fresh samples.

Fig. 3e illustrates the ion transport process in the neutral water rich polymer network: hydrated ions migrate in the aqueous phase, and the fixed charge in the polyelectrolyte can further regulate the ion concentration and mobility [[43], [44], [45], [46], [47]]. Conjugated polymers take a different route - electron transport by delocalized carriers along a continuous conjugated path, while carbon fillers need to form an electron percolation network. After the go in the PDA Gly PVA composite is reduced, the electronic conductivity is improved and the flexibility is maintained [48]. However, Fig. 4a is not suitable for supporting this conclusion - it shows the electro biological adhesion interface and its tissue adhesion mechanism, rather than the conductivity data of RGO PVA [49].

**Fig. 4:**
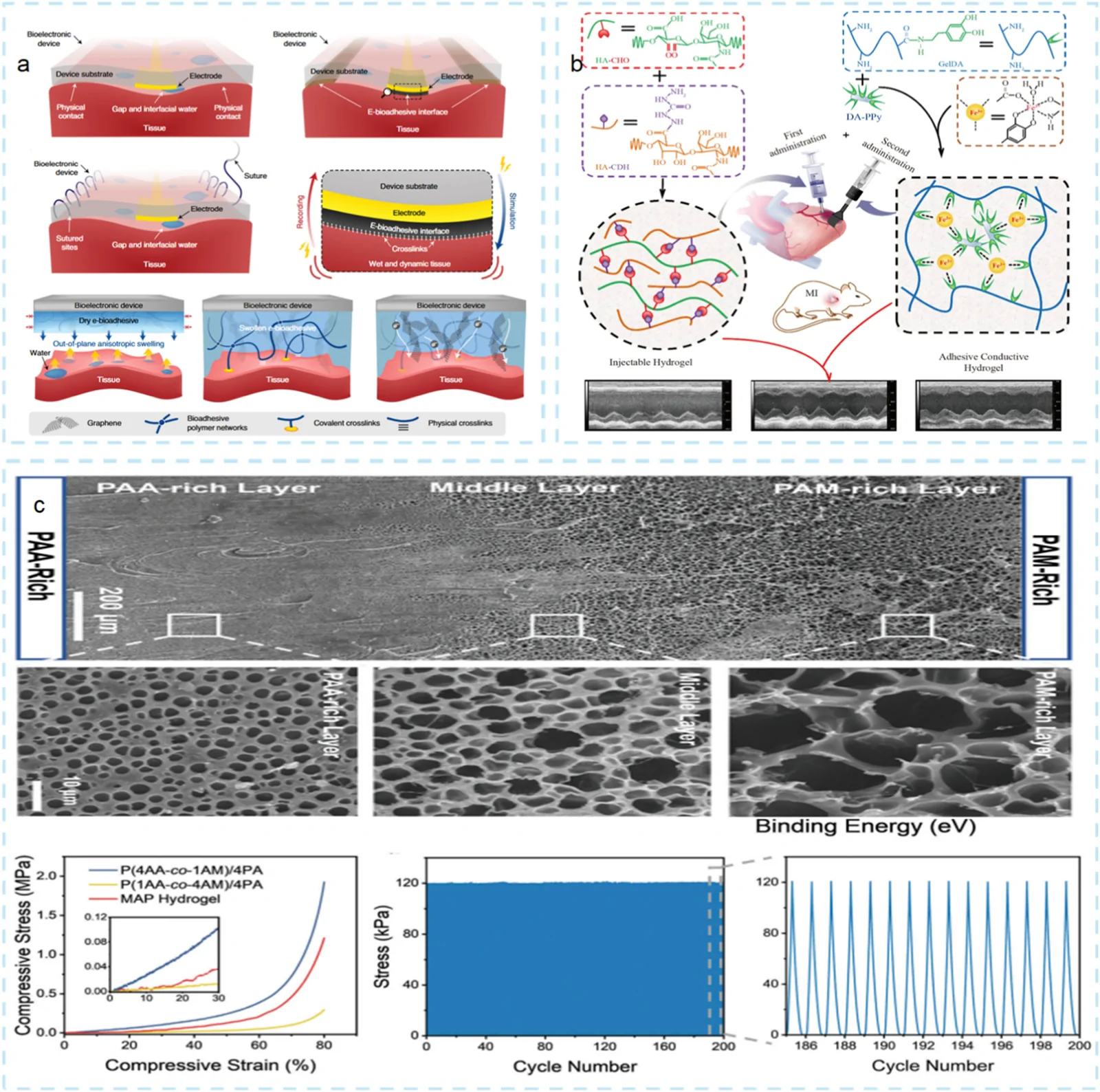
(a) Physical attachment and underlying mechanisms of bioelectronic devices to biological tissues. Copyright 2021, Springer Nature. (b) Combination of an adhesive hydrogel patch and an injectable hydrogel for the treatment of myocardial infarction. Copyright 2020, American Chemical Society. (c) SEM image of a freeze-dried MAP hydrogel cross-section from the PAM-rich side to the PAA-rich side, along with the compressive properties and fatigue resistance of the MAP hydrogel. Copyright 2025, Wiley-VCH.

### 2.4 Mechanical matching and tissue integration

Whether the implant can be stably integrated with the living nerve tissue for a long time is the final question to be answered in the above material design. The physical basis of the head is not complicated here - the mechanical properties of the implant material must be in line with the soft nerve tissue to some extent [50]. The modulus of traditional rigid electronic devices usually falls on the order of GPA, while the modulus of soft tissues such as brain and spinal cord is only about 1–100 kPa, with a gap of about three to six orders of magnitude. When the two sides are together, the biological interface will inevitably produce sustained fretting and local stress concentration. Microglia are awakened, chronic inflammation is induced, glial scars and fibrous capsules are laid out, the impedance of the electrode tissue interface rises all the way, and the signal gradually attenuates. The worst case is that the device simply fails.

Beyond the step of static adhesion, what is more critical now is to make the material design keep up with the multiple mechanical parameters of nerve tissue - elastic modulus, viscoelasticity, toughness, fatigue threshold, which are indispensable. Only by realizing dynamic matching in these dimensions can a long-term stable neural interface be considered as a real foundation. Once the mechanical fretting at the interface is pressed down, the foreign body reaction can be effectively suppressed, and the continuous and high fidelity neural communication is thus guaranteed at the physical level [51].

Precise molecular design and topology engineering are pushing the hydrogel system with tissue like mechanical properties to the front stage. For example, inspired by the hierarchical structure of "soft on the outside and hard on the inside" of nerve bundles, researchers came up with a Janus gradient structure conductive hydrogel [52,53]. It relies on the programmable gradient polymerization strategy to grow a non-uniform mechanical distribution with hard inside and soft outside - the inside is dense and tough, the modulus is about MPa, the conductive network is hidden here, and the robustness of the electrical path depends on it; The exterior is much softer, and the modulus falls to kPa, so it is not a problem to fit the nerve adventitia or cerebral cortex. This continuous transition from "hard core" to "soft shell" can just disperse and dissipate the interface stress, and the occurrence rate of mechanical damage during implantation is at least not higher than that of traditional materials [40,54,55].

Mechanical matching may reduce interfacial micromotion, but biological integration must be supported by model-specific histology and longitudinal electrical data rather than inferred from softness alone. Fig. 4a illustrates an electrical bioadhesive strategy for attaching bioelectronic devices to wet tissues [49]. Fig. 4b is a cross-domain myocardial-infarction example in which an adhesive conductive patch was coadministered with an injectable hydrogel [56]; it illustrates an assembly principle but is not direct evidence for peripheral-nerve repair or chronic neuromodulation. Accordingly, neural claims should rely on neural implantation models with quantitative glial, fibrotic, impedance, noise, and signal-stability outcomes.

Gradient network design can distribute interfacial stress and broaden pressure-response ranges, but the chemistry and application must be identified correctly. In the MAP hydrogel shown in Fig. 4c, PA denotes phytic acid, not polyampholyte. Phytic acid induces microphase separation and reinforcement in the PAA-rich region, while acting predominantly as a plasticizer in the PAM-rich region; the resulting porosity and modulus gradient was developed for an iontronic pressure sensor [57]. This wearable-sensor result is a cross-domain material-design analogue and should not be presented as direct neural-interface evidence.

## 3 Translational frontiers: from fundamental research to clinical application

### 3.1 Peripheral nerve repair and regeneration

The reason why conductive hydrogel has attracted much attention in peripheral nerve interface is largely due to its flexibility and hydration state, which help to improve conformal contact. However, it should be pointed out that Fig. 5a itself does not provide direct evidence of peripheral nerves. The left part shows the application of partially reduced graphene oxide/polydopamine/polyacrylamide hydrogel in cell stimulation and implantable bioelectronics [58], and the right part shows the gelatin micro topology of gold nanorods for myocardial tissue arrangement [59]. These interdisciplinary examples illustrate the design idea of electrical and topological clues, and cannot be used to support the conclusion of chronic peripheral nerve safety or effectiveness.

**Fig. 5:**
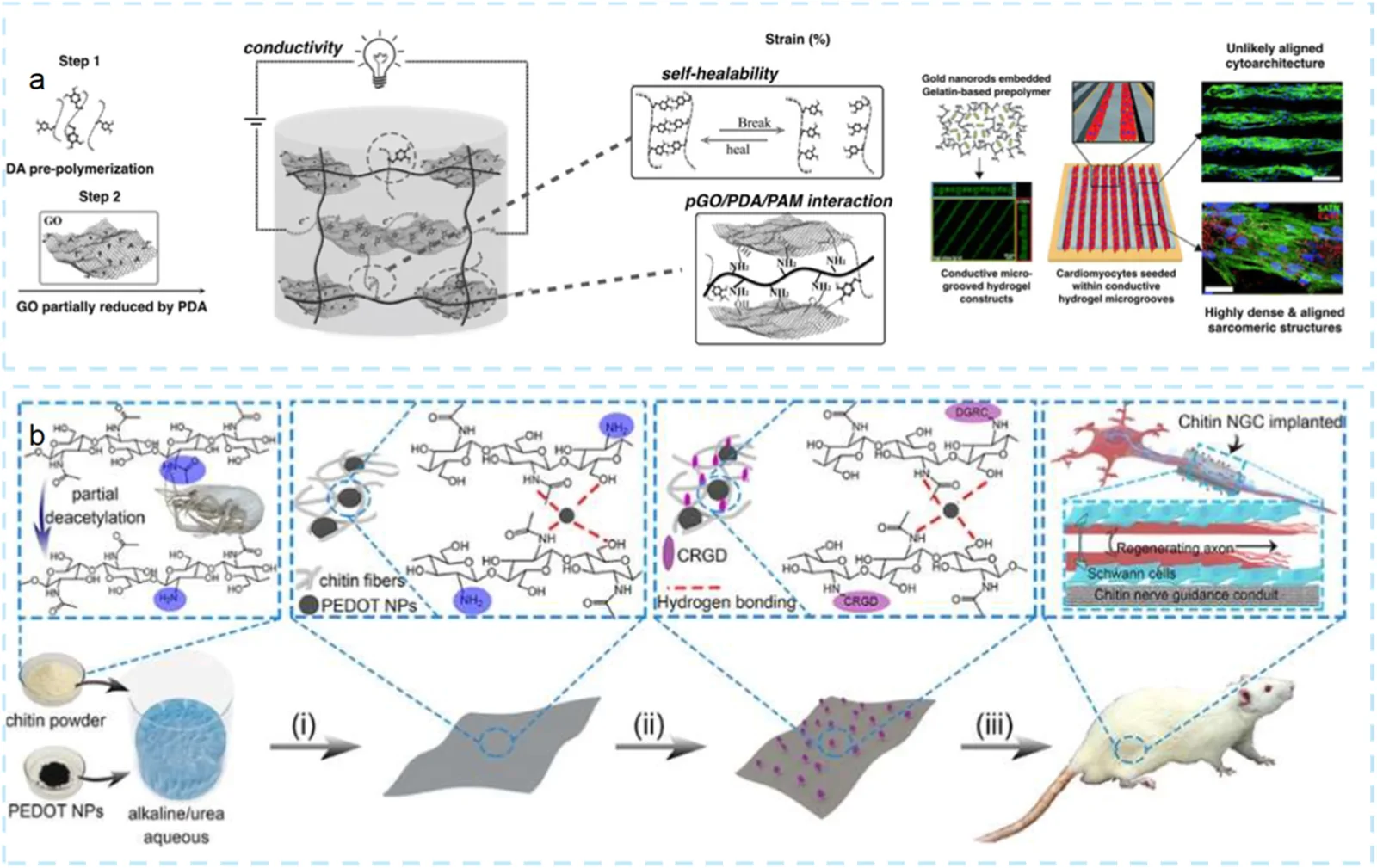
(a) Partially reduced graphene oxide composite ECHs constructed using polydopamine-crosslinked polyacrylamide. These ECHs provide biomimetic topographical and electrical cues in vitro, leading to the formation of highly aligned cellular constructs with tissue-level functionality. Copyright 2017, Wiley-VCH, and Copyright 2017, Royal Society of Chemistry. (b) Preparation of chitosan blended with PEDOT nanoparticle (NP) hydrogel, followed by surface modification with the cell-adhesive peptide CRGD to obtain ChT-PEDOT. The resulting material was then implanted into a rat sciatic nerve defect to evaluate its regenerative capacity. Copyright 2021, American Chemical Society.

Fig. 5b is different. It directly presents a peripheral nerve regeneration platform: chitin (CHT) hydrogel is combined with PEDOT nanoparticles and modified by cell adhesion peptide Cys Arg Gly ASP (CRGD), named CHT -PEDOT-P, which is used for bridging repair of rat sciatic nerve defect model [60]. This work does support the conclusion of nerve conduit regeneration, but it should not be confused with chronic electrical nerve regulation or autonomous closed-loop control.

The bioactivity effect should be attributed to the specific components, rather than the general label of "conductive hydrogel". Xuan et al. We synthesized an injectable hyaluronic acid-based self-healing network (termed hAspy) by grafting cystine and pyrrole-1-propionic acid onto hyaluronic acid, followed by coordination with Fe^3+^ ions. In rsc96 Schwann cells, the material changed the il-17ra/IL-17 signaling pathway and increased the expression of myelin related markers; After injection into the crush injury site of sciatic nerve in rats, myelin regeneration and functional recovery were improved 30 days later [54]. In another study, Yao et al. Filled the 3D printed PCL catheter with bisphosphonic acid saline gel containing Mg^2+^release. Primary rat dorsal root ganglion (DRG) culture showed that Mg^2+^was associated with PI3K/Akt activation and sema5b; After implantation of 10 mm rat sciatic nerve defect, axonal regeneration, myelin sheath regeneration and functional recovery were enhanced at 12 weeks [61]. Fig. 7b shows a different system containing mg^2+^: the adaptable alg mg/SF interpenetrating network hydrogel developed by Gao et al. Promoted Schwann cell and macrophage infiltration, axon ingrowth, myelin sheath regeneration and functional recovery within 8 weeks in 10 mm rat sciatic nerve defect [62]. This alg mg/SF platform is not the same system as the PCL/ha PAM mg catheter of Yao et al. And neither of these two studies can be used to support PEDOT: PSS mediated conduction mechanism.

The architecture shown in Fig. 6a is based on the original study of Hou et al., In 2026. They trained the mechanics of pva/gelatin double network film containing PEDOT:PSS, induced the arranged fiber like surface, loaded it to achieve NGF release, folded it into a catheter and fixed it to the nerve with chitosan suture adhesive. This study evaluated the directional growth and differentiation of PC12 cells, as well as the rat sciatic nerve defect model, and reported the early activation of calcium signaling and PPAR pathway, accompanied by axonal regeneration, myelin regeneration and functional recovery [63]. Other original studies evaluated different systems: Zhang et al. Used silk fibroin/GelMA inverse opal tube, filled with PNIPAM, PEDOT:PSS and NGF, and used ultrasound to trigger NGF release in PC12 cells and 8-week rat sciatic nerve defect model [64]; Deng et al. Evaluated the chitosan/conductive hydrogel double-layer catheter loaded with 7,8-dihydroxyflavone in the rat sciatic nerve defect model [65]; Wang et al. Developed an adhesive ECM/oxidized polysaccharide/PEDOT:PSS dressing loaded with indole-3-propionic acid for peripheral nerve repair of immune regulation [66]. Therefore, these platforms should be separately discussed and should not be combined into the same type of catheter.

**Fig. 6:**
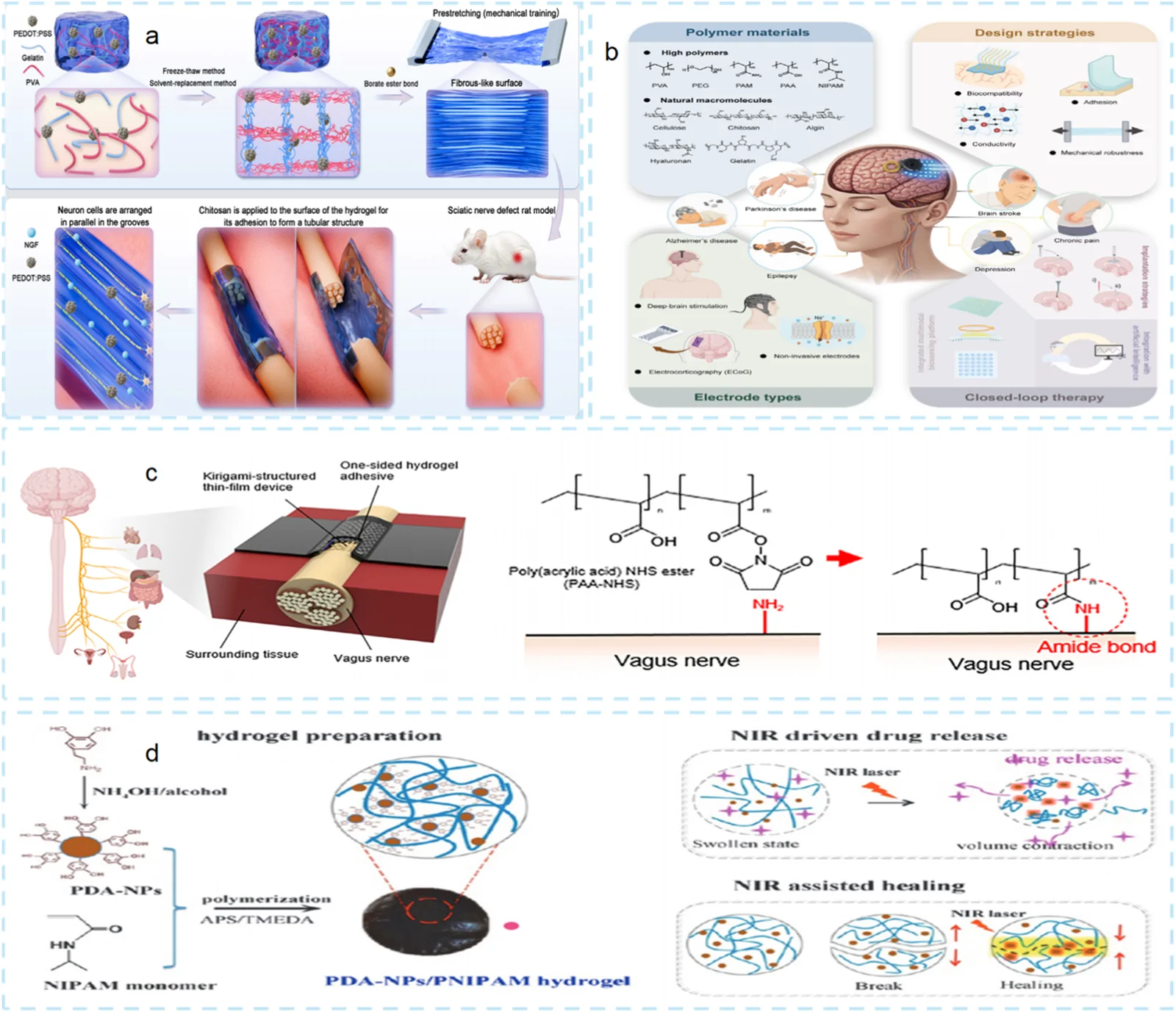
(a) PVA/gelatin/PEDOT:PSS topological nerve-guidance conduit generated by mechanical training and evaluated in PC12 cells and a rat sciatic-nerve defect model. Copyright 2026, Tsinghua University Press. (b) Overview of hydrogel-based brain–computer interfaces, including materials, electrode designs, closed-loop therapy concepts, and applications. Copyright 2024, OAE Publishing Inc. (c) Hydrogel-integrated kirigami electrode for cuff-free vagus-nerve implantation. Copyright 2025, Wiley-VCH. (d) General, non-neural PDA-NP/PNIPAM NIR-responsive drug-release and self-healing material. Copyright 2016, American Chemical Society.

**Fig. 7:**
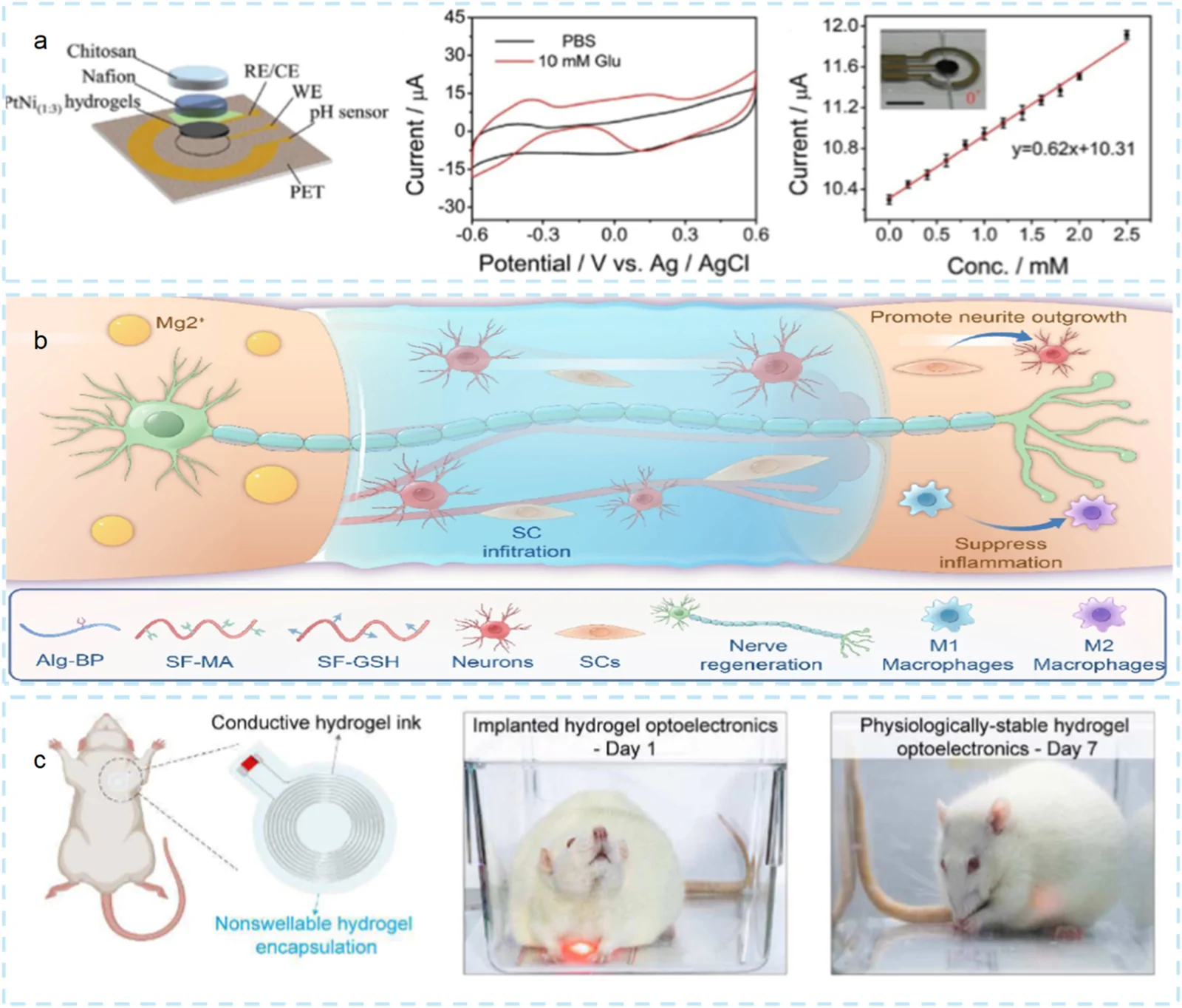
(a) PtNi(1:3) sweat-glucose sensor (module example, not a neural implant). Copyright 2023, Wiley-VCH. (b) Alg-Mg/SF peripheral-nerve repair conduit. Copyright 2024, Elsevier BV. (c) 3D-printed anti-swelling hydrogel optoelectronic implant, days 1 and 7. Copyright 2026, Springer Nature.

In the peripheral nerve area, the application of hydrogel bioelectronics is spreading a continuous functional spectrum. One is a high fidelity neural regulatory interface for chronic disease management, and the other is a multifunctional tissue regeneration platform for structural damage repair. The two lines are advancing in parallel, and the treatment of peripheral nervous system (PNS) is slowly shifting from traditional non-specific intervention to precise, individualized and functional repair [28,41,[67], [68], [69], [70]].

Although peripheral-nerve hydrogel interfaces have produced encouraging regenerative and neuromodulatory results, the evidence does not show that dynamic bonds or adhesion prevent chronic compression. Direct evidence is limited to specific devices and durations: the ICAA-C interface showed smaller impedance drift and less tissue injury than a sutured cuff over 4 weeks [24], and a cuff-free hydrogel-adhesive microelectrode reduced dependence on circumferential nerve isolation in short-term vagus-nerve experiments [71]. Neither study directly measured chronic circumferential pressure or established long-term preservation of fascicular conduction. The 8-week rat sciatic-nerve result for a viscoelastic multilayered graphene-hydrogel membrane is indirect evidence from a different material and geometry [39]. It supports the plausibility that viscoelastic conformity can reduce a foreign-body response, but it cannot be used to claim that dynamic networks as a class avoid compression. The remaining hypothesis is that swelling-controlled dynamic or adhesive networks may reduce suture tension and fixation trauma. Testing that hypothesis requires longitudinal measurements of swelling, interface pressure, displacement, nerve-conduction velocity, axonal/myelin histology and pain or motor function in anatomically relevant chronic models, including large animals before clinical extrapolation.

### 3.2 Brain-computer interfaces (BCI) and central neuromodulation

Brain computer interface (BCI) and neural regulation technology cannot bypass a core pursuit - to establish a long-term, stable and high-fidelity two-way information exchange with the central nervous system (especially the cerebral cortex). Traditional metal or silicon-based microelectrode arrays have large stiffness and high modulus. After implantation, acute and chronic immune reactions follow, and the signal attenuates severely with time. The flexible materials such as conductive hydrogel are different. It imitates the mechanical and biochemical microenvironment of brain tissue relatively well, and is bringing the interface from "intrusive" to "integrated". Therefore, the lifelong stable BCI interface has more possibilities.

At present, researchers are looking at interface materials with super flexible mechanical properties and intrinsic bio-adhesive properties, and want to achieve minimally invasive and solid integration on the surface of the cortex or deep brain [26,72]. Polydopamine (PDA) hydrogel system is a noteworthy example. Taking the bionic adhesion and conductivity of PDA as an example, the researchers built a super flexible (modulus as low as kPa) conductive hydrogel electrode. Due to the intrinsic wet adhesion, these devices can be attached to the undulating gyrus and sulcus surface without suture. The conformal contact is good, the electrical contact is stable, but the invasive pressure is very low. This "super soft adhesion" approach will at least not bring too much surgical trauma and mechanical injury; The surface itself has good biocompatibility. The activation of microglia and glial scar wrapping can be inhibited to a certain extent, which reduces the risk of immune rejection after long-term implantation, and a stable long-term neural signal acquisition can be considered as a foundation [73].

Pushing BCI towards multimodal and high-dimensional information decoding has also become an important trend in this field. Hydrogels with high transparency and high conductivity are just in use [74,75]. Such materials have little loss of visible light and specific optical bands, and can be compatible with two-photon calcium imaging, photogenetic stimulation or local blood flow monitoring while recording high-throughput electrophysiological signals (such as ECoG of cortical EEG). With the combination of electrical and optical modes in real time, the ability to analyze the coding mechanism of neural groups and loop dynamics has reached a new level. Researchers can connect the high spatial-temporal resolution of neuroelectrical signals with the activity of specific cell types or vascular dynamics, and the interpretation of neural information will be deeper. This set of integration ideas provides a tool basis for the next generation of high-dimensional, high-throughput BCI systems.

The application layout of hydrogel in BCI field is now pushing forward along two parallel paths. One focuses on mechanics and Interface Engineering, pursues deep integration with brain tissue, and relies on it to support the stability of long-term signal acquisition and stimulation; The other is the collaborative design of optoelectronic properties of materials, which goes beyond the traditional single electrical mode and pulls up the dimension of neural information acquisition and decoding accuracy. The two roads lead to the same direction - BCI technology is slowly moving from laboratory prototype to long-term implantable system with more clinical practicability (Fig. 6b) [9,[75], [76], [77]].

Although the extreme softness of the cortical hydrogel electrode is an advantage in reducing the mechanical mismatch, it has brought some troubles in practical operation. Devices with high moisture content are often difficult to implant, and it is difficult to locate and maintain geometric accuracy; Swelling may also change the electrode size or tissue contact state. Chronic performance is also prone to be limited-interface delamination, conductive layer damage, packaging or interconnection failure, and biological reactions that still exist even if gliosis is reduced may drag down. These failure modes are also different between the ECoG array on the cortical surface and the penetrating intracortical probe. Therefore, longitudinal impedance, signal-to-noise ratio, functional channel survival rate, size change and quantitative histology should be included in the report of chronic research, and acute signal quality should not be the only focus. A rabbit ECoG study over 550 days showed that the hydrogel based cortical recording can indeed be maintained for a long time on a specific platform [78]; The comparison of pedot/CNT and PEDOT:PSS coatings over a period of four months shows that the coating chemistry and chronic interface evolution will bring quite different long-term results [42]. The evidence obtained from a certain cortical architecture should not be directly extended to all BCI formats.

### 3.3 Spinal cord modulation

The spinal cord, which serves as the regulatory hub between the central relay and peripheral effectors, is indispensable for the treatment of chronic pain, the regulation of organ function and the recovery of motor function [51,[79], [80], [81]]. However, the mechanical dynamics of this tissue are substantial - the spinal cord will deform when the spine flexes and stretches - the structure is also complex, the functions of different regions are different, and the organization is fragile. Compared with brain computer interface, the requirements for mechanical adaptability, spatial resolution and long-term biocompatibility of implantable devices will not be easier or even more stringent. Conductive hydrogel offers several relevant material advantages: viscoelastic programmable, strong deformation ability, and tacit integration with the organization. It is therefore emerging as an important interface material for addressing these challenges (Fig. 6b). gives an overview of the brain computer interface (BMIs) based on hydrogel, which systematically combs common polymer materials, design strategies for enhancing electrode functions, electrode configurations, closed-loop treatment systems, and applications in the treatment of a variety of brain diseases [74,82].

In the research of spinal cord regulation, the development of high-density flexible electrode array which can dynamically fit the surface of spinal cord has become the core direction. Tendon structure water gel design brings a good idea. Inspired by this, researchers made an anisotropic conductive hydrogel, which is specially used for the spinal cord interface. This material has good tensile properties in a specific direction and can adapt to the periodic deformation of the spinal cord; In the vertical direction, the conductivity and modulus remain high, and the electrical isolation and structural stability between electrode channels will not be compromised. With this "shape adaptive" mechanical compliance, the electrode array can be closely attached to the spinal cord in physiological activities such as respiration and movement, and the long-term stable recording of sensory and motor nerve signals can be guaranteed. Microfluidic channels have also been integrated. Drugs (such as neurotrophic factors or anti-inflammatory agents) can be delivered locally synchronously - electrical stimulation and drug delivery together form an "electrical drug" collaborative platform, which provides another way for precise functional intervention and repair after spinal cord injury.

Autonomic nerve regulation. The vagus nerve is thin and moving, and it is also close to the large blood vessels. The geometric and mechanical constraints are quite specific. Park et al. Integrated paa-nhs/pva hydrogel adhesive with kirigami microelectrode with a thickness of about 3 μ m and stuck it on the vagal nerve separated from the cervical part of the rat without circumferential isolation. Under 0.4 Ma stimulation, the C-fiber evoked compound action potential with a peak value of about 1500 μV can be stably elicited (Fig. 6c) [71]. Terutsuki et al. Embedded PEDOT modified polyurethane (pedot-PU) into the stress programmed PVA bilayer structure, which can self close and wrap the nerve under the pressure of about 200 PA. In the acute cervical vagal nerve experiment in pigs, 30 Hz biphasic stimulation can induce reversible bradycardia [83]. These two studies do support the feasibility of sleeveless or self closing hydrogel assisted VNS, but they can not be generalized as "any dynamic covalent hydrogel can prevent chronic compression injury".

The current evidence mainly supports the acute or short-term conformal adhesion of the vagus nerve interface, which is not enough to explain the effect of mesenchymal stem cells/hydrogel in repairing vagus nerve injury or long-term prevention of compression injury [71,83]. Those conclusions cannot be inferred from the relevant studies of spinal cord or frostbite. Fig. 6d is reserved, but it is only a universal design example of response materials-pda-np/pnipam hydrogel shows near infrared controlled pulse release, drive and light assisted self repair in non neural scenes [84], which is not directly related to vagus nerve repair or chronic nerve regulation. The chronic transformation verification still depends on the measured data: cuff pressure, impedance drift, induced response stability, axon and myelin integrity, as well as macrophage and fibrosis reactions, which need to be measured directly in the clinical implantation cycle.

During the process of breathing and movement, the spinal cord interface always bears repeated flexion, extension, torsion and multiaxial loads. The risks of fatigue, motion artifacts, displacement, and interconnect failure arise from this. Penetrating devices still have to shoulder a lot - both to deal with implantation trauma and to maintain long-term position stability. Although the anisotropic CNT-pva microelectrode showed the recording ability for eight months, the current evidence is all from the mouse model; The quantitative histological data of GFAP/Iba-1 are also mainly concentrated in the early window of 3 to 30 days after implantation [85]. Go back, multi axis fatigue test should be done, longitudinal impedance, signal-to-noise ratio, channel survival rate, displacement measurement and late quantitative histopathology should also be followed, and finally the verification should be completed on the clinical relevant anatomical scale.

## 4 Technical challenges and future perspectives

In a word, hydrogel bioelectronics has great potential in the field of chronic neuromodulation, but there are still many hurdles to be overcome before it can really enter the stage of large-scale clinical application and long-term reliable use. How to improve the intrinsic performance of materials, how to straighten out the integration of devices and systems, and how to lay the clinical transformation path are intertwined, which constitute the main bottlenecks restricting the development of this field [22,28,[86], [87], [88], [89]].

### 4.1 Long-term stability and degradation control

Implantable devices must balance functional stability with degradation kinetics, but Fig. 8 combines distinct evidence types. In Fig. 8a, the left portion shows MorphE, a viscoplastic PEDOT:PSS/glycerol neural interface that accommodated growth of rat peripheral nerves while preserving conduction velocity over 8 weeks [90]. The right portion shows a fully implantable bioresorbable cardiac pacemaker and its dissolution, a cross-domain cardiac example [91]. Fig. 8c shows lipase-mediated degradation and bovine-serum-albumin release from Lam-GMA-crosslinked polyacrylamide hydrogels [92]. The cardiac and drug-delivery panels illustrate design concepts only and are not direct evidence for degradation-controlled chronic neural bioelectronics.

**Fig. 8:**
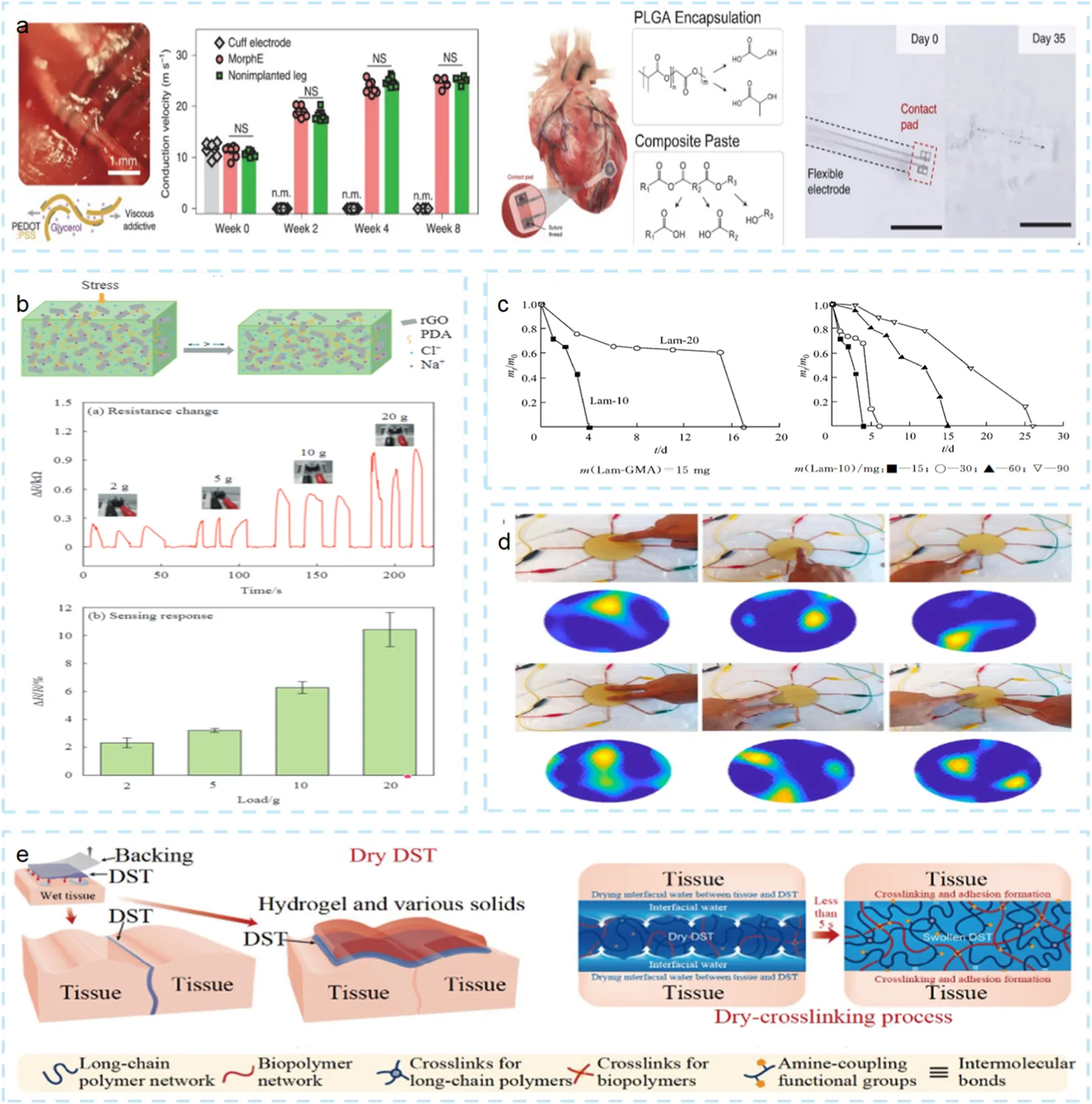
(a) A highly biocompatible and bioresorbable cardiac pacemaker and its degradation process upon immersion in PBS solution. Copyright 2020, Nature Biotechnology and Copyright 2021, Springer Nature. (b) PDA/rGO-10 hydrogel in NaCl solution, its electricity generation mechanism, and the resistance change (ΔR) and pressure sensing response of the hydrogel under different loadings. Copyright 2025, Science Press. (c) Degradation curves of hydrogels crosslinked with Lam-10. Copyright 2014, Editorial Department of Journal of Functional Polymers. (d) Conductive images showing a single finger in contact and multiple fingers in contact simultaneously. Copyright 2024, American Chemical Society. (e) Schematic illustration of an instantly adhesive dry double-sided tape (DST) and its dry crosslinking mechanism. Copyright 2019, Springer Nature.

For natural biodegradable hydrogels such as silk fibroin (SF), the biocompatibility and cell support ability are good, but the degradation kinetics has been difficult to control [[93], [94], [95]]. The ideal degradation rhythm must roughly match the "golden time window" of nerve regeneration. In the early stage of regeneration, the scaffold should support the physical structure, maintain the electrical pathway, and guide the axon directional growth; After the initial reconstruction of the neural network, the material should be degraded in a timely and orderly manner to give space to new tissues [64,[94], [95], [96], [97], [98]]. If the degradation is too fast-faster than the axon extension speed - the electrode function will be lost, the structure will collapse, and the regeneration process will be broken; If the degradation is too slow or incomplete, the foreign body will remain, and the chronic inflammation or fibrosis will wrap up, but the functional recovery will be inhibited. The impact of local accumulation of degradation products on the microenvironment (pH, ion concentration) also deserves more attention.

Therefore, the key to overcome this bottleneck is to go in the direction of the next generation of intelligent hydrogels and develop on-demand, programmable degradation characteristics. For future material design, it is advisable to introduce external stimuli (near-infrared light, ultrasound, magnetic field) or internal biological signals (specific enzymes, pH, and reactive oxygen levels) to make a switch to trigger degradation. For example, by embedding enzyme sensitive cross-linking in the gel network or encapsulating responsive microcapsules, the material can be selectively and gradually decomposed after nerve regeneration has passed the key nodes. This "self timing" or "signal response" degradation strategy can at least make the material come out of the passive biological erosion entity and become a role that actively adapts to and participates in the biological process. The real "diagnosis and treatment integration" bioelectronic implants may lay the foundation on this point [[99], [100], [101], [102], [103], [104]].

### 4.2 Signal fidelity and anti-interference

The internal environment of chronic implantation has always been complex. It is necessary to record the nerve electrical signals for a long time and keep the noise low enough, which represents a major technical challenge in precise neuromodulation. Most of the root causes of signal quality degradation are biological interference and interface instability. The initial implantation trauma will induce acute inflammation, and the subsequent continuous foreign body reaction will lead to glial hyperplasia and dense fibrous capsule, and the high impedance biological insulation layer at the electrode tissue interface will become thicker and thicker [[105], [106], [107], [108]] Micromotion and stress caused by respiration, heartbeat, and body movement also remain challenging. The interface contact impedance fluctuates back and forth, and motion artifacts are often mixed into the signal.

Biological claims should be separated by inferential level. Direct evidence is confined to the tested composition, stimulus, cell type, anatomical model and observation period. In the BaTiO_3_/collagen system, ultrasound-driven piezoelectric stimulation increased calcium signaling and mitochondrial bioenergetics through PIEZO1 in astrocytes and PIEZO2 in Schwann cells and supported repair in the corresponding animal models [67]. This result demonstrates glial mechanotransduction that is specific to both composition and stimulus.; it does not establish a universal glia-regulating property of conductive hydrogels and does not directly demonstrate microglial reprogramming. Indirect analogy comes from studies in which mechanical matching, viscoelasticity or related physical cues altered cell behavior or reduced a foreign-body response [28,39,[109], [110], [111], [112]], but those studies differ in material chemistry, target tissue, stimulus and endpoint. The broader proposition that other dynamic, adhesive or conductive hydrogels can actively regulate astrocytes, Schwann cells or microglia therefore remains a hypothesis requiring cell-type-specific markers, causal perturbation, dose-response analysis and chronic functional/histological validation. Fig. 7c is a separate anti-swelling, biphasic conductive hydrogel manufacturing platform [113] and is retained only as engineering evidence, not as visual evidence of glial phenotype regulation.

Research ideas are shifting from simply optimizing the electrical properties of materials to designing interfaces that can actively regulate local biological reactions. Mechano bioactive hydrogels provide a possible way to combine tissue matched viscoelasticity with stimulation induced cell signal transduction [28,67,[109], [110], [111], [112]]. The BaTiO_3_/collagen platform in astrocytes and Schwann cells provides direct evidence for the piezo dependent response mediated by ultrasound [67]; Fig. 7c shows another swelling resistant biphasic conductive hydrogel for three-dimensional printing of implantable bioelectronic devices [113]. The latter reflects the ability of preparation and device integration, rather than the general mechanism of glial cell regulation. These two types of evidence need to be clearly distinguished in use.

In the future, the research on improving the quality of neural signals will increasingly fall on the system level design. The closed-loop feedback system deserves more efforts to make the real-time monitoring of interface impedance, the adaptive adjustment of stimulus parameters, and the on-demand release of anti-inflammatory drugs linked together; Advanced signal processing algorithms should also be connected to eliminate physiological noise at the two levels of material source and data analysis. Long term stable and high signal-to-noise ratio neural interaction cannot escape the cross fusion of materials science, cell biology and computational neuroscience [[114], [115], [116], [117]].

### 4.3 Wireless operation and miniaturization

Wireless power transfer, wireless telemetry, energy harvesting, and self-powered sensing are separate engineering functions. Fig. 8b shows a PDA/rGO hydrogel swollen in NaCl solution that generated small electrical outputs under externally applied loads and exhibited pressure-dependent resistance [118]. The study supports bench-scale energy harvesting and self-powered pressure sensing; it does not demonstrate wireless power transfer, an implanted power link, or delivery of a clinically relevant neural-stimulation energy budget.

Self-powered hydrogel devices based on piezoelectric, triboelectric, thermoelectric, or ionic-gradient mechanisms may eventually supplement low-power sensing [[119], [120], [121], [122]]. For PDA/rGO-10, the directly reported evidence is pressure-induced output in NaCl solution (approximately 0.40 μA and 0.54 mV under a 10 g load in 0.05 mol L^−1^ NaCl) together with resistive pressure sensing [118]. Those values should not be extrapolated to continuous implant powering, wireless charging, neural stimulation, or autonomous closed-loop therapy without a complete power budget, rectification and storage circuitry, encapsulation data, in vivo validation, and stimulation-safety testing.

On the side of miniaturization, the goal is not complicated -- on the premise of maintaining electrical and biological properties, the size of devices should be reduced as much as possible, but the function integration should be improved. The hydrogel itself is soft, and micro nano processing technologies such as lithography, roll to roll printing and micro molding can be used. Therefore, high-density and micron scale electrode arrays can be made; Heterogeneous integration with silicon-based chips can also spell out a "rigid flexible" hybrid micro system. But as the size shrinks, trouble follows. The uniformity of conductive hydrogel in micron scale is not easy to control, and the packaging reliability in long-term implantation environment is confusing. The design and integration of miniature wireless communication antennas also remain challenging. To deal with this, materials science, microelectronics engineering and biomedicine have to requires close interdisciplinary collaboration.

In a word, the two issues of wireless and miniaturization are closely intertwined with each other, which is essentially a system level engineering topic. Looking ahead, if we can make a small, all wireless and long-term stable bioelectronic device like "nerve dust", the chronic regulation of nerve activity will probably be insensitive, accurate and lasting [123,124].

### 4.4 Integration and intelligentization of closed-loop systems

Several recent original studies have given different levels of evidence at the system integration level. The paacp hydrogel constructed by Chu et al. Takes pam-paa as the matrix and adds 0.33 wt% polydopamine to modify carbon nanotubes, with a conductivity of 9.52 s m ^−1^ and tissue adhesion, thus making a sutureless sciatic nerve cuff, which can realize two-way electrical communication and integrate into the nerve repair system controlled by real-time feedback [22]. Qin et al. Developed an α-helix polypeptide crosslinked supramolecular hydrogel probe, which recorded the local field potential in the prefrontal cortex of rats for 16 weeks. At the same time, it can carry out in situ nerve regulation and record induced electric activity in the chronic neuropathic pain model [125]. The latter shows the ability of long-term two-way recording and stimulation, rather than the autonomous perception decision execution closed loop. Fig. 7a shows a wearable sweat glucose sensor chip based on PtNi (1:3) hydrogel, which integrates pH sensing and wireless reading functions [126]. It's a metabolic sensor, not an implantable neural interface, so it is not suitable for direct evidence of closed-loop neural regulation. The complete closed-loop hydrogel neural regulation still needs to clearly report the perceived biomarkers, decision-making rules, stimulation renewal strategies, delays, safety boundaries and treatment outcomes, which cannot be inferred only by multi-functional integration.

Traditional open-loop neuromodulation uses preset parameters despite time-varying neural states. A future closed-loop system must explicitly connect sensing, a decision rule, and a controlled intervention while reporting latency and safety limits. Hydrogel interfaces may support this integration, but multifunctionality alone is not evidence of a closed loop. Fig. 7a demonstrates wireless readout in a wearable metabolic sensor [126], not an implanted sensing-decision-stimulation neural system; it is therefore retained only as a module-level analogue.

Fig. 8d does not show an implantable flexible circuit. It shows tactile localization by a multimodal artificial-skin array during single- and multiple-finger contact [127]. This cross-domain demonstration supports the feasibility of spatially resolved hydrogel sensor arrays, but it provides no direct evidence for implanted neural recording, inflammatory monitoring, drug release, adaptive stimulation, or closed-loop neuromodulation. Those system-level claims require a defined neural signal, decision rule, actuator, latency, safety limit, and in vivo therapeutic outcome.

Hydrogel itself provides an interesting physicochemical platform for intelligent system. By introducing the stimulus responsive components (temperature sensitive, enzyme sensitive and photosensitive groups), the hydrogel can become an actuator with its own "computing" ability. For example, when a hydrogel containing glucose oxidase encounters an increase in local glucose concentration, the degree of swelling or conductivity will change, and the intensity of electrical stimulation attached to its surface can be automatically adjusted - thus, there is an autonomous feedback mechanism for the coupling of neural activity and metabolic state.

Looking at artificial intelligence, the trend of integration has been relatively clear. By deploying lightweight AI models in vivo or on near end devices (such as wearable devices), it is possible for the system to learn and predict the dynamic patterns of individual neural activity. From "reactive" treatment to "pre adaptive" regulation, this leap is should be presented as a development objective rather than an established capability [128,129].

Therefore, the future closed-loop system will probably go in this direction-bio friendly interface, multimodal sensing, edge intelligent computing, adaptive execution, and multiple functional modules will be deeply integrated into an organic whole. As the core interface between devices and biological tissues, hydrogel design should not only focus on the material itself, but also include the system level integration requirements and intelligent potential. If this set of approaches is adopted, neural regulation can be changed from "manual setting" to "autonomous optimization", and personalized dynamic treatment of chronic nervous system diseases can also be regarded as a landing direction.

### 4.5 Scalable manufacturing and clinical translation pathways

To turn hydrogel bioelectronic devices from a breakthrough prototype in the laboratory into a reliable device for clinical use, there are still two hurdles to cross. One is large-scale and repeatable manufacturing, and the other is systematic clinical transformation. This work is not only the engineering amplification of materials and processes, but also involves scientific supervision, standardized production and clinical verification. It is a complex system engineering.

On the manufacturing side, laboratory synthesis and microfabrication must be translated into high-throughput, reproducible, GMP-compatible processes. Hydrogel performance is sensitive to humidity, temperature, batch composition, sterilization, and packaging. Fig. 8e shows a dry double-sided tape that rapidly bonds wet tissue to tissue or devices through interfacial water removal followed by covalent crosslinking [130]. It is a cross-domain assembly strategy rather than a validated manufacturing solution for chronic neural implants; device-specific fatigue, sterilization, shelf-life, extractables/leachables, and package-reliability testing remain necessary.

Let's talk about the clinical transformation path. This path needs to be paved by systematic and rigorous preclinical and clinical research. The cycle of safety and effectiveness evaluation in animal models is much longer than that of typical academic research-a few years is the minimum. The biocompatibility, functional stability, aging and failure modes of devices in complex physiological environment must be fully understood. More importantly, the study was designed to meet the real clinical needs. Take Parkinson's disease deep brain stimulation or spinal cord injury repair. How to determine the device performance indicators (service life, power consumption, signal bandwidth), how to select the animal model, and how to evaluate the treatment end point are all in line with the clinical diagnosis and treatment guidelines of the target disease. Materials scientists, engineers, and clinicians have to sit together and cooperate in depth since the day the project is launched.

Whether regulatory approval can really overcome this barrier depends largely on whether the regulatory system has been actively understood and adapted in the early stage. This matter cannot be delayed until later. Researchers should be able to understand the specific requirements of the target market (such as the FDA of the United States and the nmpa of China) for products such as "implantable nerve stimulator" or "tissue engineering scaffold" in a forward-looking manner, and incorporate the considerations of quality control and risk management into the design process in advance. The road from laboratory samples to products on the market is really difficult, but we must go through it. Interdisciplinary and interdisciplinary in-depth cooperation is indispensable. The whole chain of basic innovation, process development, clinical validation, registration and declaration must be linked together. Only in this way can hydrogel bioelectronic technology really release the potential of improving human health.

#### 4.5.1 NAM-enabled development and validation pathway

When we consider NAMs (new approach methodologies) in the development process of conductive hydrogel neural interface, we might as well organize them into a decision gate that passes in sequence, rather than scattered single detection. After a round of screening based on human cells, the candidate formula will enter the interface model at the tissue level, controlled toxicity and degradation test, and computer optimization. The evidence generated by these steps will eventually point to those questions that still need chronic in vivo verification to be answered [11,12].

In the door of material screening, neural cells and neural organs derived from human IPSC can be used to compare the effects of cell compatibility, maturity of neurons and glial cells, network activity, stiffness, conductivity, cross-linking density, residual chemicals and sterilization methods. The integration of organoid and bioelectronics has been able to achieve electrophysiological records across tissues or for several months [131,132]. Therefore, it is possible to screen at the functional level, and it is no longer just to watch the cell live or die.

At the door of the interface, the compartmentalized neural chip MPs can reproduce the orderly structure of axon Schwann cells and output clinical related conduction results; Three-dimensional microelectrode arrays enable direct functional coupling with neural structures [[133], [134], [135]].

The problem of long-term failure should be investigated by real-time and accelerated aging/degradation models, which combine hydrolysis, enzymatic hydrolysis and oxidative exposure with cyclic mechanics and electrical loading. The obtained dynamics and failure modes can be coupled to finite element or multi physical field models of field, current density, Joule heat, stress-strain, swelling and mass transfer; These frameworks can handle complex nerve electrode geometry and stimulation conditions [136]. Table 4maps each type of NAMs to a specific development decision, and indicates the animal experiment parameters that should be refined (see Table 5).

**Table 4:** NAM-enabled development and validation pathway for conductive-hydrogel neural interfaces.

Development question	NAM platform or model	Minimum readouts and decision gate	Development question
Material screening	Human iPSC-derived neural cells, 3D neural cultures, and neural organoids	Viability, neuronal/glial phenotype and maturation, network electrophysiology, and responses to stiffness, conductivity, crosslink density, dopants, and sterilization; down-select only biologically and functionally acceptable formulations.	Removes poorly tolerated or electrically unstable materials before device fabrication and animal implantation.
Interface toxicity and functional coupling	Compartmentalized nerve-on-chip and other neural microphysiological systems under direct contact, extract exposure, perfusion, stimulation, and mechanical loading	Axon/neurite integrity, Schwann-cell and microglial response, myelination, conduction velocity, compound action potential, impedance, stimulation threshold, and stimulation-related injury; reject interfaces that impair local neural function.	Identifies local interface hazards and optimizes material-device architecture using tissue-level functional endpoints.
Chemical toxicity	Mechanism-based in vitro toxicity assays using the final sterilized device, extracts, residual monomers/crosslinkers, conductive fillers, leachables, and degradation products	Dose-response cytotoxicity, oxidative stress, inflammatory cytokines, genotoxicity where relevant, and exposure margins; rank hazards and define worst-case extraction or exposure conditions.	Supports chemical-risk assessment and prevents avoidable exploratory animal toxicity studies.
Long-term degradation and failure	Real-time and accelerated hydrolytic, enzymatic, and oxidative degradation models combined with cyclic mechanical/electrical loading and, where useful, perfused MPS	Water uptake, mass and molecular-weight loss, pH/osmolality, byproduct release, modulus, adhesion, fatigue, impedance drift, charge-injection stability, and delamination; define the dominant failure mode and time window.	Sets chronic in vivo duration, sampling points, failure criteria, and histological/chemical endpoints.
Animal-study optimization	Finite-element and multiphysics models calibrated with the experimental NAM data	Electric field, current density, Joule heating, stress/strain, swelling, mass transport, and degradation kinetics with sensitivity and uncertainty analysis; select geometry, stimulation limits, and worst-case configurations.	Refines species, target anatomy, duration, endpoints, stopping rules, and sample-size assumptions for focused confirmatory animal studies.

**Table 5:** summarizes the representative preclinical evidence of conductive hydrogel nerve interface in chronic implantation, and also reveals the step of transforming to large animal model. At present, there is still a gap to be filled.

Neural target	Representative hydrogel interface	Species and model category	Implantation duration	Longitudinal electrical evidence and NR fields	Histological evidence and NR fields
Cerebral cortex	CHIP-based all-organic ECoG array	Rabbit (nonrodent; not classified here as a conventional large-animal model)	>550 days	Stable cortical recording; normalized SNR remained >0.94 of the day-1 value at day 550 in a representative rabbit [78]. NR: chronic stimulation threshold, quantitative device migration, and cohort-level failure rate.	Histological evaluation indicated a limited foreign-body response [78]. NR: standardized quantitative histopathology matched to all chronic electrical time points.
Spinal cord	Anisotropic CNT-PVA hydrogel microelectrode	Mouse (rodent)	8 months	Spontaneous single-unit activity remained recordable under anesthetized and conscious conditions at 8 months [85]. NR: longitudinal numerical SNR and impedance trajectories, chronic stimulation threshold, and device migration.	GFAP decreased over time and Iba1 remained relatively consistent, but the reported histology was mainly limited to 3-30 d [85]. NR: quantitative histopathology matched to the 8-month electrical endpoint.
Peripheral nerve	ICAA-C hydrogel-cuff interface	Rat vagus nerve (rodent)	4 weeks	Only a small impedance change was observed; the conventional sutured cuff control increased by approximately 9.5-fold [24]. NR: chronic recording SNR, stimulation-threshold trajectory, and device migration beyond 4 weeks.	NFM, S-100, and Iba1 were evaluated quantitatively at 4 weeks, with less tissue injury and inflammation than in the cuff control [24]. NR: histology beyond 4 weeks and material biodistribution or clearance.

NR, not reported in the cited primary study or not reported at the specified chronic time point. Rabbit is classified here as a nonrodent model, not as a conventional large-animal model. In this review, clinically scaled large-animal evidence refers to studies in pigs, sheep, dogs, or nonhuman primates with anatomy and surgical deployment relevant to the intended human device.

**Table 6:** Target-specific surgical, mechanical, and chronic-evaluation requirements for hydrogel neural interfaces.

Comparison dimension	Cerebral cortex	Spinal cord	Peripheral nerve
Typical implantation route	Craniotomy followed by surface ECoG placement; penetrating interfaces require insertion into cortical tissue	Laminectomy with epidural/subdural placement, or penetrating intraspinal implantation	Surgical nerve exposure followed by cuff wrapping, conformal attachment, or in situ hydrogel injection
Dominant mechanical challenge	Brain pulsation and electrode-brain micromotion	Repeated flexion, extension, torsion, respiration, and locomotion-induced multiaxial deformation	Nerve sliding, stretching, and repeated deformation caused by limb or organ motion
Major surgical risk	Cortical trauma, insertion injury, and incomplete conformity to gyri/sulci	Cord injury, device migration, and restricted implantation space	Compression injury, suture trauma, and disturbance of small-diameter nerves
Hydrogel design priority	Very low modulus, low swelling, conformability, and low impedance; penetrating devices must also retain insertion compatibility	Anisotropic/multiaxial fatigue resistance, dimensional stability, and migration resistance	Strong but atraumatic wet adhesion, anti-swelling behavior, stretchability, and cyclic fatigue tolerance
Key chronic endpoints	SNR, functional channel survival, impedance drift, dimensional stability, and glial response	SNR/impedance during motion, migration, channel survival, and mechanical fatigue	Stimulation threshold, nerve conduction, impedance, selectivity, and compression-related histology
Representative failure modes	Interfacial delamination, swelling, channel loss, and gliosis	Fatigue, migration, motion artefact, and interconnect failure	Slippage, swelling-induced compression, chronic cuff deformation, and loss of intimate coupling

The evidence obtained from these checkpoints should be treated as a whole evidence package. As long as the front pass has not been passed, we can not take the next step; The results of NAMs should in turn guide the species selection, target site, observation duration, sample size setting, chronic endpoint index, termination rules and device configuration in the worst case of animal experiments. In this way, NAMs can indeed replace some exploratory screening steps and significantly reduce and refine the subsequent confirmatory animal studies, and can also reduce and optimize confirmatory animal experiments. However, the current NAMs cannot completely simulate human scale surgical operations, systemic immunity and clearance, vascular remodeling, or the fretting between devices and tissues over the years [11,12].

#### 4.5.2 Gap between long-term preclinical evidence and large-animal translation

The preclinical study of conductive hydrogel nerve interface is currently transitioning from short-term proof of concept experiments to chronic implantation. However, the verification duration and scale between different nerve targets are not small. In terms of cortex, Zhu et al. Tested an all organic cortical electroencephalogram (ECoG) array on a rabbit, a non rodent model. Its core is the microstructure of conductive hydrogel interface permeability network (chip). The longest record was more than 550 days. On the 550th day, the normalized signal-to-noise ratio (SNR) of one representative animal remained above 0.94 (relative to the first day). Histological examination also showed that the foreign body reaction was relatively mild [78]. In the spinal cord, the anisotropic CNT-pva microelectrode can still record the discharge activity of single neuron eight months after implantation in mice, but the quantitative histological data of GFAP and Iba1 are mainly concentrated at the time point of 3 to 30 days after implantation [85]. In the peripheral nerve, the impedance of ICAA-C vagus nerve interface changed little after four weeks, while the impedance of sutured commercial sleeve increased by about 9.5 times; NFM, S-100 and Iba-1 analysis results show that the tissue damage and inflammatory reaction caused by the hydrogel interface are lighter [24]. These studies do provide important support for the feasibility of different targets, but none of them alone can produce a complete chronic transformation data package.

Within the research scope included in this structured narrative review, we have not found any conductive hydrogel nerve interface that can meet the following conditions at the same time: it is implanted in the conventional large animal model (pig, sheep, dog or non-human primate) for at least six months, and the longitudinal electrophysiological records and matched quantitative histopathological evaluation are carried out simultaneously. The ECoG study on rabbits does provide valuable long-term non rodent data, but rabbits are not included in the conventional large animal model here, and that work also fails to cover human scale anatomy, surgical approach, fixation method, wire routing, chronic fretting or device failure and other practical problems. Therefore, this gap is more suitable to be regarded as a clear preclinical milestone, rather than generally attributed to the "long transformation cycle".

#### 4.5.3 Target-specific surgical and device requirements

In the case of clinical transformation, it is impossible to rely on a set of general hydrogel optimization strategies. The cortex, spinal cord and peripheral nerve differ greatly in surgical approach, physiological movement mode, acceptable fixation methods, and the consequences of swelling or mechanical constraint. Therefore, the index of "softness" alone is far from enough to constitute a complete design criterion. Modulus, viscoelastic relaxation, swelling behavior, adhesion, fatigue resistance, mechanical mechanism during implantation, and electrical stability all have to be adapted one by one according to the target anatomical site and the specific operation method.

What the cortical surface interface values is conformal bonding ability and minimizing the strain caused by fretting. For devices penetrating the cortex, it is additionally required to be accurate enough when implanted and stable after being in place. If the spinal cord interface has to bear multiple axial loads repeatedly, the fatigue resistance and dimensional stability cannot be sloPPy, and the fixation method must be firm and as far as possible without additional trauma-the long-term implanted CNT-pva spinal cord microelectrode just reflects these needs [85]. On this side of the peripheral nerve interface, we should not only avoid circumferential compression, but also maintain stable electrical coupling on the small diameter nerve that continues to move. The injectable and adhesive ICAA-C strategy shows an idea: reduce the dependence on tight mechanical fixation by swelling control and interstitial adhesion [24]. However, the safety of chronic compression still needs to be verified by direct measurement.

#### 4.5.4 Clinical-trial status and regulatory pathway

On September 2nd, 2026, we searched clinicaltrials.gov with the key words "conductive hydrogel" or "hydrogel electrode" or "hydrogel nerve interface" or "hydrogel bioelectronics") and (nerve regulation or "nerve stimulation" or "nerve recording" or "brain computer interface" or "peripheral nerve" or "spinal cord") [137]. As long as it is a human intervention study, the conductive hydrogel interface or device implanted or surgically placed for nerve recording, therapeutic nerve stimulation, nerve regulation or brain computer interface will be evaluated, regardless of the recruitment status. Non human studies, observational records, non-conductive hydrogels, drug delivery or tissue repair hydrogels without bioelectronic function, non neural devices, and surface electrodes were excluded. The retrieval returned a record (nct06421753), which is about the comparison of surface dry electrode and hydrogel electrode in functional electrical stimulation of quadriceps femoris. Because it does not involve the implantable nerve interface, it is also excluded. Therefore, we have not found the type that meets the conditions in the experiments of public registration. It should be noted that this conclusion is limited to the publicly available registration information, and does not mean that there is no unpublished development project or confidential exemption application activities for test devices [138].

The regulatory classification depends on the intended use, anatomical target, implantation duration, therapeutic function and the overall risk level of the device, rather than the hydrogel material itself. In the United States, implantable peripheral nerve stimulator for pain relief (product code gzf; 21 CFR 882.5870) is a class II device, which usually takes the 510 (k) path [131]; While some fully implantable spinal stimulators are classified as class III and require pre market approval (PMA) [139]. Other spinal cord stimulation product codes may correspond to different paths, so a device cannot be generally labeled with "class II" or "class III". Research on implantable devices with significant risks needs to comply with the IDE framework of 21 CFR Part 812 [138]; FDA guidelines for implantable BCI devices also provide relevant recommendations for non clinical testing and ide/clinical research design [140]. Domestically, the classification guide of nmpa in 2026 classified invasive or implantable BCI equipment as class III, with classification code of 12-00 [141]; For the test of high-risk class III devices, additional nmpa clinical trial approval may be required [142].

#### 4.5.5 Manufacturing, sterilization, and quality-control barriers

On September 2nd, 2026, we searched clinicaltrials.gov with the key words of ("conductive hydrogel" or "hydrogel electrode" or "hydrogel nerve interface" or "hydrogel bioelectronics") and (nerve regulation or "nerve stimulation" or "nerve recording" or "brain computer interface" or "peripheral nerve" or "spinal cord") [137]. As long as it is human intervention research, the evaluation is that the clinical transformation of the implanted or surgically placed conductive hydrogel interface or device has not been realized yet, which cannot be simply attributed to the fact that this field is not "new". In fact, there are several types of specific evidence missing and the project gap has not been filled.

First, we have not found any research that can simultaneously meet the following conditions in large animal models (pigs, sheep, dogs or non-human primates) on a clinical scale: implantation for at least six months, and complete recording of longitudinal signal-to-noise ratio or impedance trajectory, stimulus threshold changes, device displacement evaluation, and matched quantitative histopathology. Therefore, the long-term ECoG study on rabbits can only be regarded as evidence of non rodents, and should not be directly renamed as large animal validation.

Second, the stability at the material level has not yet been reliably translated into the verified service life of devices. Bench aging and immersion experiments can help us screen materials, but it is far from enough to prove that it can last for several years in a human body with quite complex mechanical and chemical environments.

Third, the consistency of manufacturing batches is still a bottleneck at present. Water content, crosslinking density, PEDOT:PSS doping state, CNT or MXene dispersion, ion composition, and the interface between hydrogel and electrode - any link fluctuation may cause obvious deviation in the electrical and mechanical properties of the batch.

Fourth, sterilization, packaging and storage cannot be treated as back-end details, but must be included in the overall verification of the final device. The effects of irradiation, ethylene oxide sterilization, dehydration and storage conditions on the mechanical properties, adhesion ability, chemical structure and electrochemical properties of hydrogels are closely related to the specific formula, and should be evaluated one by one by experiments. FDA guidelines also repeatedly emphasize that the sterilization process should be verified, and the impact of sterilization on device performance and biocompatibility should also be considered [143] (see Table 7).

Fifth, the long-term material safety must be evaluated for the final device, and cannot be concluded only by the label of body composition, antioxidant activity, short-term cell survival rate or "biocompatibility". Here, we make a distinction: direct evidence refers to the experimental data of the same final formula, the same anatomical target, the same exposure route, the same endpoint and duration; Indirect analogy refers to evidence from related materials, different cell types, different targets or device configurations; Hypothesis means that a benefit or risk has only been proposed at the conceptual level and has not been tested in the relevant chronic models. Table 8 maps these classifications to major chemical and biological risks. A complete risk management framework should integrate formula specific chemical characterization, extractable/extract analysis, toxicological risk assessment, and chronic tissue device endpoints under the framework of ISO 10993 and current FDA recommendations [144,145].

**Table 7:** Regulatory documents and pathways relevant to conductive-hydrogel neural interfaces.

Regulatory body/framework	Key document or pathway	Relevance to conductive-hydrogel neural interfaces
FDA	Implanted Brain-Computer Interface (BCI) Devices for Patients with Paralysis or Amputation - Non-clinical Testing and Clinical Considerations (2021) [140]	Provides concrete recommendations for nonclinical testing and IDE/clinical-study design for implanted BCI devices
FDA/21 CFR	21 CFR Part 812 - Investigational Device Exemptions [138]	Applies to investigations of significant-risk devices; implanted neural devices presenting potential serious risk generally require an IDE before study initiation
FDA	Implanted peripheral nerve stimulator for pain relief, product code GZF, 21 CFR 882.5870, Class II, 510(k) [146]	Illustrates that an implanted neuromodulation device may follow a Class II pathway depending on intended use and device classification
FDA	Totally implanted spinal-cord stimulator for pain relief, product code LGW, Class III, PMA [139]	Illustrates a higher-risk neuromodulation pathway requiring PMA; classification is product-code and indication specific
FDA/ISO 10993-1	Use of International Standard ISO 10993-1: Biological evaluation of medical devices - Part 1 (2023) [144]	Supports risk-based biological evaluation of long-term tissue-contacting materials, including consideration of processing, constituents, degradation products, and leachables
NMPA	Guidance Principles for Classification and Definition of Brain-Computer Interface Medical Devices (2026) [141]	Invasive/implantable BCI medical devices are managed as Class III, classification code 12-00
NMPA	Approval framework for clinical trials of higher-risk Class III medical devices [142]	Certain high-risk Class III device clinical trials require national regulatory approval before initiation
NMPA	Revised Good Manufacturing Practice for Medical Devices (2025; effective 1 Nov 2026) [147]	Establishes quality-management requirements relevant to scalable, reproducible manufacturing and process control

**Table 8:** Material-specific safety risks, present evidence boundaries, and minimum evaluation requirements for conductive-hydrogel neural interfaces.

Risk source	Main concern and exposure route	Direct evidence in the reviewed studies	Evidence boundary	Minimum evaluation and follow-up requirements
Residual monomers and oligomers	Unreacted acrylamide, acrylic acid, methacrylate or NHS-bearing species may diffuse from the hydrated network and cause local cytotoxicity, neurotoxicity or sensitization.	Representative studies generally report final-gel viability or short-term histology, but do not provide batch-specific residual-monomer concentrations [[20], [21], [22], [23], [24]].	Indirect only: acceptable cell viability does not demonstrate chemical absence or a safe chronic exposure margin.	Measure conversion and targeted residues by HPLC/LC-MS/GC-MS; perform exhaustive extractables testing and toxicological risk assessment on final, sterilized and aged devices; include acute cell assays and chronic implantation endpoints matched to intended use [144,145].
Crosslinkers, initiators and catalysts	APS/TEMED, photoinitiators, aldehydes, coupling reagents and coordination ions may remain reactive or generate toxic by-products.	Direct evidence is formulation-specific and is usually limited to cytocompatibility or short animal observations; complete residual profiles are rarely reported.	Hypothesis requiring testing: network formation or washing is often assumed to remove these species, but this cannot be inferred without analytical recovery data.	Quantify parent compounds and reaction products; validate washing, sterilization and shelf-life effects; use polar and non-polar extracts, dose-response cytotoxicity, sensitization/irritation where relevant, and subchronic-to-chronic local histology [144,145].
Carbon nanotubes (CNTs)	Potential release of free or fragmented CNTs, macrophage uptake, oxidative stress, persistence and migration from the implant site.	PAACP was evaluated in a rat sciatic-nerve system [22]. CNT-PVA microelectrodes recorded for up to 8 months, but GFAP/Iba-1 histology mainly covered 3-30 days [85].	Direct device function is not equivalent to chronic filler safety; long-term release, biodistribution and clearance remain unquantified.	Characterize CNT loading, dispersion and bound/free fraction; quantify released material during cyclic ageing; assess neural and macrophage responses, local nerve conduction, quantitative histology and, where release is plausible, organ distribution at staged chronic endpoints including ≥ 6 months for permanent devices.
MXene	Oxidation, sheet fragmentation and release of Ti-containing or surface-termination species may alter ROS generation, immune response and electrical performance.	The MXene/PEDOT:PSS/TA-containing ICAA interface showed stable function and reduced tissue injury over 4 weeks in a rat vagus-nerve model [24].	Direct for that final formulation and 4-week model only; it does not establish chronic MXene biodistribution, clearance or class-wide safety.	Track oxidation state, particle size and soluble/particulate release during PBS/serum ageing; use ICP-MS and microscopy where appropriate; test ROS, neural/glial and macrophage responses, local histology and biodistribution over durations matched to a permanent implant.
PEDOT:PSS	PSS-rich fractions, low-molecular-weight additives, dedoping products or polymer fragments may leach; electrical overdrive may change local chemistry.	T-ECH, TN-H and ICAA provide formulation-specific cytocompatibility, electrochemical or short-term in vivo evidence [20,24,122]; several regeneration platforms also contain PEDOT:PSS [63,64,66].	Indirect for chronic neural safety: most studies do not combine long-term chemical release profiles with quantitative neural histology.	Measure extractables, TOC and targeted additives before and after sterilization/electrical cycling; monitor dedoping and degradation under worst-case stimulation; pair chemical data with neural viability, electrophysiology and quantitative 3-, 6- and 12-month histology when intended for permanent use.
Tannic acid (TA)	Dose-dependent protein binding, oxidation products and diffusible polyphenols may affect cells, coagulation or local redox balance.	TA is one component of ICAA, for which 4-week device-level compatibility was reported [24]; the biological contribution of TA was not isolated.	Indirect: safety of the composite cannot be assigned to TA alone, and a beneficial antioxidant assay does not substitute for release-dose characterization.	Quantify free TA and oxidation products during ageing; establish release-dose-response relationships in neural, glial and immune cells; assess hemocompatibility when blood exposure is plausible and include chronic local histology.
Nanoparticles and metal ions	BaTiO3, PDA nanoparticles and released Fe^3+^/Mg^2+^ or other ions may cause particle persistence, ion imbalance, ROS or off-target immune effects depending on dose and localization.	BaTiO3/collagen plus ultrasound directly altered astrocyte and Schwann-cell signaling in its tested repair models [67]; Fe^3+^- and Mg^2+^-containing regenerative systems provide separate, composition-specific evidence [54,61,62].	No class effect can be inferred across particle chemistry, dose, target tissue or stimulus. Mechanistic benefit does not itself demonstrate chronic systemic safety.	Measure particle size, dissolution and ion release by validated analytical methods; determine local dose, biodistribution and organ retention; test ROS, immune activation, genotoxicity where indicated, and quantitative local/systemic histology over the intended exposure period.
Degradation products	Acidic/basic fragments, oligomers, particles or ions may change pH, osmolality, inflammation and electrical/mechanical integrity as the network degrades.	Reviewed studies commonly report mass loss, swelling or retained function, whereas identities, concentrations and toxicity of intermediate products are seldom resolved [64,[93], [94], [95], [96], [97], [98], [99], [100], [101], [102], [103], [104]].	Mostly hypothetical until the actual product spectrum and clinically relevant exposure are measured; nominal biodegradability is not proof of harmless resorption.	Combine real-time and accelerated hydrolytic, oxidative and enzymatic ageing with LC-MS/GC-MS/SEC/ICP-MS as appropriate; test worst-case product mixtures at measured concentrations and align degradation, nerve regeneration and histology time courses.
Extractables and leachables	Additives, solvents, surfactants, adhesives, packaging constituents and sterilization-generated species may be released from the final assembled device.	A comprehensive final-device extractables/leachables profile is not reported for the representative neural-interface systems reviewed here.	Evidence gap: material-purity statements and single-component tests cannot substitute for final finished-device chemical characterization.	Apply ISO 10993-18-aligned exhaustive extraction using justified polar/non-polar media, analytical evaluation thresholds and targeted elemental analysis; identify and quantify constituents and perform ISO 10993-17 toxicological risk assessment on sterilized and aged devices [140,145].
Inflammation and histological observation duration	Acute injury can evolve into macrophage/microglial activation, astrogliosis, fibrosis, axonal loss, demyelination or compression neuropathy after early time points.	Model-specific evidence includes 4-week ICAA vagus-nerve histology [24], 8-week graphene-membrane sciatic-nerve inflammation [39], early 3-30-day GFAP/Iba-1 assessment for the 8-month CNT-PVA recording study [85], and long-term rabbit ECoG evidence in a different cortical platform [78].	Direct only for each stated material, anatomy, endpoint and duration. Cross-model comparison is indirect and cannot establish lifetime safety or absence of chronic compression.	Use blinded quantitative Iba-1/CD68, GFAP, fibrotic-capsule, NFM/S100/myelin and axon metrics with nerve conduction and device displacement/pressure where relevant; include acute, subchronic and ≥ 6-month endpoints for permanent implants, plus terminal organ histology when material release is plausible.

We have adopted a relatively conservative approach in labeling the evidence labels in Table 8. The direct evidence will not be used beyond the formula, anatomical site, end point and duration verified by it; Indirect analogy can only explain a certain possibility, and cannot be regarded as confirmatory evidence; As for the hypothesis, it cannot be presented as an established biological benefit. NR means not reported. The test plan should be designed according to the expected tissue contact type, implantation duration, degradation behavior, and the sterilization or aging state in the worst case, rather than all hydrogels follow the same process.

Sixth, the failure of the device is not necessarily due to the hydrogel itself. Most of the time, it is the hydrogel metal interconnection, package interface, tissue adhesion interface, wire transition, connector or package itself that have problems first. The dry-type double-sided tape mentioned in Fig. 8e may be useful as an assembly principle [130], but to use it in chronic nerve implants, targeted verification must be done for fatigue, sterilization, shelf life, extractable/extractable substances and interface reliability.

In a word, the clinical transformation should be done as the development of devices for specific targets, rather than the natural extension of the study of hydrogel materials. A feasible path should at least include: validation on clinical relevant anatomical scales, preset chronic electrophysiological and histological endpoints, manufacturing specifications and batch release standards, sterilization and aging validation, risk-based biological evaluation, and alignment with the applicable FDA or NMPA regulatory path as soon as possible.

## 5 Conclusions

Hydrogel bioelectronics is entering a key node to accelerate the transformation from laboratory innovation to clinical application. This review summarizes the main progress in this field, from which we can see that with the help of bionic design, multi-level structure engineering and interface functionalization, conductive hydrogels have gradually grown into a kind of "intelligent" interface materials that can dynamically adapt to nerve tissue, electrophysiological regulation and biological activity induction, which can take into account. Its core advantages have been gradually recognized in the chronic nerve regulation: the mechanical properties can be adjusted and matched, and the foreign body reaction after implantation can be reduced to a certain extent; The interface adhesion and anti swelling ability are online, and the stability of long-term electrical contact will not easily go wrong; There is a natural affinity with biological tissues, so there is a relatively favorable microenvironment for nerve repair and functional regulation. Together, these characteristics have pushed hydrogel bioelectronics to many application scenarios, such as peripheral nerve repair, brain computer interface, spinal cord injury regulation, and so on. In the face of traditional rigid electronic devices, hydrogel bioelectronics also shows some unique confidence.

In short, there are still many systemic challenges to move from laboratory to clinical application. The long-term stability and controllable degradation of implants, the maintenance of high fidelity signals in complex physiological environments, and the landing of wireless and miniaturized closed-loop systems are the most striking. From experimental prototypes to reliable medical devices, standardized and large-scale manufacturing processes and clear clinical transformation paths are all thresholds that must be crossed. Materials science, flexible electronics, neural engineering and clinical medicine need to be deeply integrated together. In the future, more efforts should be made on the robust hydrogel network, mixed ionic-electronic transport architecture, standardized preparation process, and systematic transformation evaluation framework. Only when these problems are solved one by one can hydrogel bioelectronics really take a step forward from the level of proof of concept.

The future development will probably focus more and more on two directions: one is to improve the intelligence of the material itself, and the other is to seek breakthroughs in system integration. If the intelligent hydrogel that can respond to multiple bio electro mechanical signals is integrated with sensing, feedback and executive functions, the adaptive and personalized neural regulation system is expected to become a reality. Once the challenges of long-term stability, wireless power supply, and large-scale manufacturing are successfully addressed, this technology has completed a substantial leap from cutting-edge concepts to clinical therapy. Nervous system diseases such as Parkinson's disease and chronic pain can probably wait for a new generation of precise, minimally invasive and lasting treatment options.

Accordingly, the translational endpoint proposed in this review is not an immediate jump from an optimized hydrogel to large-animal implantation. It is a NAM-enabled sequence in which neural organoids screen material formulations and network function; nerve-on-chip/MPS and targeted toxicity assays resolve interface injury; degradation and accelerated-ageing models identify long-term chemical, mechanical, and electrical failure; and computational models select device geometry, stimulation limits, and the focused questions to be tested in animals. Used as fit-for-purpose decision gates, these approaches can make chronic in vivo studies smaller, more targeted, and more informative while preserving animal confirmation for surgery, systemic biology, and long-duration performance [11,12,[131], [132], [133], [134], [135], [136]].

## CRediT authorship contribution statement

**Chunlin Liu:** Writing – review & editing, Writing – original draft, Conceptualization. **Yukun Mao:** Writing – original draft, Conceptualization. **Zhenxing Ren:** Investigation. **Yuanhong Shang:** Investigation. **Hui Xu:** Investigation. **Wanyue Feng:** Visualization. **Jixin Zhou:** Writing – original draft. **Duo Zhang:** Writing – review & editing. **Xiang Cui:** Writing – review & editing, Writing – original draft. **Xulin Hu:** Writing – review & editing, Writing – original draft, Investigation, Conceptualization.

## Funding

This work was supported by the Sichuan Science and Technology Program [grant number 25GJHZ0372]; The State Key Laboratory of New Ceramic Materials 10.13039/501100004147Tsinghua University [KF202529]; 10.13039/501100001809National Science Foundation of China (10.13039/501100001809NSFC, #82402822); 10.13039/501100018542Sichuan Natural Science Foundation Program (General Program, #2026NSFSC0599); 10.13039/501100019014Chengdu Science and Technology Program Technology Innovation Research and Development Project (#2026-YF05-01303-SN); The central government of Sichuan Province guides the special project of local science and technology development (#2024ZYD0155); 10.13039/501100020207Health Commission of Sichuan Province Medical Science and Technology Program(#24QNMP036); Youth Innovation Project of Sichuan Medical Association (#Q2024092).

## Declaration of competing interest

The authors declare that they have no known competing financial interests or personal relationships that could have appeared to influence the work reported in this paper.

## Data Availability

Data will be made available on request.
